# Tensor decomposition-based unsupervised feature extraction applied to matrix products for multi-view data processing

**DOI:** 10.1371/journal.pone.0183933

**Published:** 2017-08-25

**Authors:** Y-h. Taguchi

**Affiliations:** Department of Physics, Chuo University, 1-13-27 Kasuga, Bunkyo-ku, Tokyo 112-8551, Japan; Nanjing Normal University, CHINA

## Abstract

In the current era of big data, the amount of data available is continuously increasing. Both the number and types of samples, or features, are on the rise. The mixing of distinct features often makes interpretation more difficult. However, separate analysis of individual types requires subsequent integration. A tensor is a useful framework to deal with distinct types of features in an integrated manner without mixing them. On the other hand, tensor data is not easy to obtain since it requires the measurements of huge numbers of combinations of distinct features; if there are *m* kinds of features, each of which has *N* dimensions, the number of measurements needed are as many as *N*^*m*^, which is often too large to measure. In this paper, I propose a new method where a tensor is generated from individual features without combinatorial measurements, and the generated tensor was decomposed back to matrices, by which unsupervised feature extraction was performed. In order to demonstrate the usefulness of the proposed strategy, it was applied to synthetic data, as well as three omics datasets. It outperformed other matrix-based methodologies.

## Introduction

In the current era of big data, it is often that massive datasets are obtained, including samples with many features. For example, a video dataset can be regarded as time points (samples) vs pixels (features). Audio files consist of time points (samples) vs amplitude (features), and sets of DNA sequences consist of individuals (samples) vs nuclear acid sequences (features). All of these are provided in the form of a matrix, whose rows and columns are features and samples, respectively (of course, rows and columns are exchangeable). Although processing massive datasets is itself problematic, integrating distinct types of datasets is even more difficult. This problem is often annotated as multi-view data processing. For example, an audio visual file can be regarded as time points (samples) vs pixels and amplitudes(both features). In this paper, I consider two types of specific multi-view data processing: one is sharing samples (hereinafter called Case I) and another is sharing features (hereinafter, called Case II). It is formally possible to deal with these two cases as unified; multiple (*m* > 0) views of data, i.e., *X*^(*k*)^, *k* = 1, … *m*, each of which is *N*_*k*_ features times *M* samples shared with multiple views (Case I), can be regarded as a (∑_*k*_ *N*_*k*_) × *M* matrix

$$\begin{array}{cc} {\left(X^{(1)T},X^{(2)T},\cdots ,X^{(m)T}\right)}^{T}, & \end{array}$$ (1)

where *X*^*T*^ is the transposed matrix of *X*, while, if *X*^(*k*)^, *k* = 1, … *m*, are *M*_*k*_ samples times *N* features shared with multiple views (Case II), can be regarded as a *N* × (∑_*k*_ *M*_*k*_) matrix

$$\begin{array}{cc} (X^{\left(1\right)},X^{\left(2\right)},\cdots ,X^{\left(m\right)}). & \end{array}$$ (2)

For both cases, however, we are not sure what will happen by simply merging distinct features as one matrix.

In order to address this problem, a variety of multi-view data processes have been proposed [1, 2]. Independent of the strategy to integrate multi-view datasets, there must be some weights attributed to each view. Since there are no *a priori* criteria to optimize these weights, some kind of artificial criteria are required. For example, if samples are classified, weights can be optimized so as to discriminate samples coincidentally from classes. Alternatively, if feature extraction is a task, weights can be optimized so as to generate the “best” features regardless of which features are considered good.

The reason weights are required for individual views is that we do not know whether the same weights are acceptable when simply creating new variables by merging or linearly combining them. Suppose $x_{ij}^{\left(k\right)}$ are the observed values attributed to the *i*th feature of the *j*th sample in the *k*th view. Generating a merged matrix is to have a matrix where $x_{ij}^{\left(k\right)}$ is placed at the (*i* + ∑_*k*′ < *k*−1_ *N*_*k*′_)th row and the *j*th column, as introduced in Eq (1) (Case I). Alternatively, a merged matrix can be generated where $x_{ij}^{\left(k\right)}$ is placed at the *i*th row and the (*j* + ∑_*k*′ < *k*−1_ *M*_*k*′_)th column, as introduced in Eq (2) (Case II).

This is not necessarily as simple as it may appear. For example, in Case I, if *N*_*k*_ varies drastically from view to view, the results may be dominated by the views with the maximum number of features. However, it is not clear if views with more features are more important. Alternatively, if the new feature $x_{ij}^{\prime }=\sum_{i,(k)}C_{i}^{\left(k\right)}x_{ij}^{\left(k\right)}$ is generated with the linear combination where $C_{i}^{\left(k\right)}\text{s}$ are coefficient, there are similar problems. If $C_{i}^{\left(k\right)}\text{s}$ do not vary dependent upon (*k*), views with more features may dominate the outcome. Reverting row and column, I will discuss Case II as well. In order to compensate for this discrepancy, each view must be weighted based on criteria which are not naturally unique. No previously proposed strategies were free from this problem.

In this paper, I propose a brand new strategy that is free from weighting views; generating tensors whose number of modes is the same as, or one greater than the number of views, and applying tensor decomposition (TD) to them. Using this implementation, I performed feature extraction (FE), which I name TD based unsupervised FE, which is extended from the recently proposed principal component analysis (PCA) based unsupervised FE [3–22].

## Materials and methods

### Converting multi-view matrices into a tensor with multiplication

If we generate a new feature with neither summation nor merging, the product for Case I—$x_{i_{1},i_{2},...,i_{m},j}=\prod_{k=1}^{m}x_{i_{k},j}^{\left(k\right)}$—can be regarded as an (*m* + 1) mode tensor. As each newly generated feature is composed of one feature from individual views, no weight is needed. Similarly, in Case II, $x_{j_{1},j_{2},...,j_{m},i}=\prod_{k=1}^{m}x_{i,j_{k}}^{\left(k\right)}$, can be regarded as an (*m* + 1) mode tensor. These tensors are hereinafter called Type 1.

Alternatively, instead of simply multiplying matrix components with the shared columns or rows, they can be summed up as follows: ${\tilde{x}}_{i_{1},i_{2},...,i_{m}}=\sum_{j}\prod_{k=1}^{m}x_{i_{k},j}^{\left(k\right)}$ (Case I) and ${\tilde{x}}_{j_{1},j_{2},...,j_{m}}=\sum_{i}\prod_{k=1}^{m}x_{i,j_{k}}^{\left(k\right)}$ (Case II). These can be regarded as *m*-mode tensors and are hereinafter called Type II. All variables associated with Type II tensors are written with tildes.

These newly generated *m*-mode (type II) or (*m* + 1)-mode (type I) tensors can be processed using any kind of tensor manipulation. For example, for a reduced number of features whose combination can express tensors, TD can be used to gain such features.

In the following subsection, I consider four combinations of types and cases, i.e., type I or II tensors for Case I or II multi-view data, case by case.

### Definition and terminology of TD

Since TD is not a popular methodology and the usage of TD for FE is rare, I will briefly introduce TD in this subsection.

TD is the expansion of tensor *x*_*n*_1_,*n*_2_,…,*n*_*m*__, *n*_*k*_ = 1, …, *N*_*k*_, 1 ≤ *k* ≤ *m* in the form

$$\begin{array}{c} x_{n_{1},n_{2},\ldots ,n_{m}}=\sum_{\ell_{1}=1}^{N_{1}}\cdots \sum_{\ell_{m}=1}^{N_{m}}G\left(n_{1},n_{2},\ldots ,n_{m}\right)\prod_{k=1}^{m}x_{n_{k},\ell_{k}}, \end{array}$$

where *x*_*n*_*k*_,*ℓ*_*k*__, 1 ≤ *k* ≤ *m*, are orthogonal matrices. Since *x*_*n*_1_,*n*_2_,…,*n*_*m*__ is as large as *G*(*n*_1_, *n*_2_, …, *n*_*m*_), this formula is clearly overcomplete and does not give unique expansion. In this study, in order to decide *G*(*n*_1_, *n*_2_, …, *n*_*m*_), *x*_*n*_*k*_,*ℓ*_*k*__, 1 ≤ *k* ≤ *m* uniquely, I employ the higher order singular value decomposition (HOSVD) algorithm [23], which has successfully used to analyse microarrays [24] previously. *G*(*n*_1_, *n*_2_, …, *n*_*m*_) is a core matrix. *x*_*n*_*k*_,*ℓ*_*k*__, 1 ≤ *k* ≤ *m*, are singular value matrices and their column vectors are singular value vectors. *G*(*n*_1_, *n*_2_, …, *n*_*m*_), having larger absolute values, has more contribution to *x*_*n*_1_,*n*_2_,…,*n*_*m*__. Since the combination of *x*_*n*_*k*_,*ℓ*_*k*__, 1 ≤ *k* ≤ *m*, associated with *G*(*n*_1_, *n*_2_, …, *n*_*m*_) to which larger absolute values were attributed contributes more collectively to *x*_*n*_1_,*n*_2_,…,*n*_*m*__, they are more likely to be associated with one another.

For type I tensors this expression is straightforward. (*m* + 1) modes correspond to *m* + 1 components, *i*_1_, *i*_2_, …, *i*_*m*_, *j* (Case I) or *j*_1_, *j*_2_, …, *j*_*m*_, *i* (Case II), respectively. On the other hand, for type II tensors, *m* modes correspond to *m* components, *i*_1_, *i*_2_, …, *i*_*m*_ (Case I) or *j*_1_, *j*_2_, …, *j*_*m*_ (Case II), respectively. Thus, singular value vectors for *j*th sample (Case I) or *i*th feature (Case II) are missing. These can be computed via ${\tilde{x}}_{\ell_{m+1}=\ell_{k},j}^{\left(k\right)}=\sum_{i_{k}}{\tilde{x}}_{\ell_{k},i_{k}}x_{i_{k},j}$ (Case I) or ${\tilde{x}}_{\ell_{m+1}=\ell_{k},i}^{\left(k\right)}=\sum_{j_{k}}{\tilde{x}}_{\ell_{k},j_{k}}x_{i,j_{k}}$ (Case II), *k* = 1, …, *m*. Thus, for type II tensor, there are *m* kinds of sample (Case I) or feature (Case II) singular value vectors in contrast to the type I tensors that have unique sample (Case I) or feature (Case II) singular vale vectors.

### The relation to HO GSVD

Higher order generalized singular value decomposition [25] (HO GSVD) is the method that corresponds to singular value vectors when TD is applied to type I tensors. As HO GSVD converts *X*^(*k*)^ = *U*^(*k*)^Λ*V*^*T*^ where *U*^(*k*)^, Λ and *V* are the *N*_*k*_ × *M* left singular value matrix, *M* × *M* eigenvalue matrix, and the *M* × *M* right singular value matrix, *U*^(*k*)^ are regarded as feature singular value matrices and *V* is regarded as a unique (common) sample singular value matrix in the present implementation.

### Synthetic dataset

The synthetic dataset used for demonstrating the usefulness of TD based unsupervised FE is defined as:

$$\begin{array}{ccc} x_{ij}^{\left(1\right)} & = & \frac{c}{2}\left(\frac{j}{M}+\sin\frac{\pi j}{M}\right)+\left(1-c\right)\varepsilon_{ij}^{\left(1\right)}\\ x_{ij}^{\left(2\right)} & = & \frac{c}{2}\left(\frac{M-j}{M}+\sin\frac{\pi j}{M}\right)+\left(1-c\right)\varepsilon_{ij}^{\left(2\right)} \end{array}$$

for 1 ≤ *i* ≤ *N*_0_. $x_{ij}^{\left(k\right)}=\varepsilon_{ij}^{\left(k\right)}$ for *N*_0_ < *i* ≤ *N*. 1 ≤ *j* ≤ *M*. $\varepsilon_{ij}^{\left(k\right)}$ obeys uniform distribution ∈ [0, 1]. Specifically, *c* = 0.8, *N* = 1000, *N*_0_ = *M* = 50.

### mRNA and miRNA expression profile

mRNA and microRNA(miRNA) expression profiles of multi-omics data were downloaded from gene expression omnibus (GEO) using GEO ID GSE28884. At first, GSE28884_RAW.tar was downloaded and expanded. For mRNA, 161 files whose names ended by the string “c.txt.gz” were used. Each file was loaded into R by read.csv command and the second column named “M” was employed as mRNA expression values. Probes not associated with Human Genome Organisation (HUGO) gene names were discarded and 13393 probes were remained. For miRNA, 161 files whose names ended by the string “geo.txt.gz” and the corresponding samples of mRNA expression which were measured were used. Each file was loaded into R by read.csv command and the second column (“Count”) was summed using the same third column (“Annotation”) values. Sum totals of less than 10 were discarded. As a result, 755 features remained. Finally, the miRNA expression profile matrix is $x_{i_{2},j}^{\text{miRNA}},1\leq i_{2}\leq 755,1\leq j\leq 161$, and the mRNA expression profile matrix is $x_{i_{1},j}^{\text{mRNA}},1\leq i_{1}\leq 13393,1\leq j\leq 161$.

mRNA expressions of epidermal growth factor (EGF) treated breast cancers were downloaded from GEO using GEO ID GSE84096. The file named GSE84096_series_matrix.txt.gz included in “Series Matrix File(s)” was downloaded. Gene expression was divided into 14 control samples and 14 EGF treated samples named $x_{i,t_{1}}^{\text{control}}$ and $x_{i,t_{1}}^{\text{EGF}}$, respectively.

mRNA expressions of vaccination experiments were downloaded from GEO using GEO ID GSE18323. Files named GSE18323-GPL570_series_matrix.txt.gz and GSE18323-GPL571_series_matrix.txt.gz included in “Series Matrix File(s)” were downloaded. As these included two distinct platforms, 22277 commonly included probes were used. Fifty eight samples annotated as “Protected group” (P) at time points T1 to T5, 52 samples annotated as “Delay group” (D) at time points T1 to T5, and 72 samples annotated as “Non-protected group” (NP) at time points T1 to T5, were used. They were named as $x_{i,t_{1}}^{\text{Pl}}$, $x_{i,t_{2}}^{\text{D}}$ and $x_{i,t_{3}}^{\text{NP}}$ respectively.

All expression profiles were standardized as ∑_*i*_ *x*_*ij*_ = 0, and $\sum_{i}x_{ij}^{2}=N$.

### PCA-based unsupervised FE

#### PCA

In contrast to the usual use of PCA, where samples are embedded, the genes were embedded in this implementation.

Suppose *x*_*ij*_s satisfy $\sum_{i}x_{ij}=0,\sum_{i}x_{ij}^{2}/N=1$ and *X* is a matrix whose elements are *x*_*ij*_. The gram matrix *G* is defined as *G* ≡ *XX*^*T*^. Eigenvectors $u_{k}={\left(u_{k1}and\ldots ,u_{kN}\right)}^{T}\text{s}$ (1 ≤ *k* ≤ min(*M*, *N*)) are then obtained as $Gu_{k}=\lambda_{k}u_{k}$, where *u*_*ki*_ is the *k*th PC score attributed to gene *i* and *λ*_*k*_s are the eigenvalues ordered as *λ*_*k*_ ≥ *λ*_*k*+1_. The *k*th PC loadings attributed to the *j*th sample *v*_*kj*_ are defined as $v_{k}=X^{T}u_{k}$, where $v_{k}={\left(v_{k1},\ldots ,v_{kM}\right)}^{T}$ because $v_{k}$ is the eigenvector of the covariance matrix *X*^*T*^ *X*, $X^{T}Gu_{k}=X^{T}XX^{T}u_{k}=X^{T}Xv_{k}=\lambda_{k}X^{T}u_{k}=\lambda_{k}v_{k}$.

#### PCA-based unsupervised FE applied to mRNA/miRNA expression

First, the five initial PC loadings *v*_*ℓ*_1_, *j*_s (for mRNA) and $v_{\ell_{2},j}^{\prime }\text{s}$ (for miRNA), 1 ≤ *ℓ*_1_, *ℓ*_2_ ≤ 5, were confirmed to have significant sample dependence (*P*-values < 0.05) with categorical regression,

$$\begin{array}{ccc} v_{\ell_{1},j} & = & C_{\ell_{1}}^{0}+\sum_{S}C_{\ell_{1},S}^{1}\delta_{Sj},\;\;1\leq j\leq 161,\\ v_{\ell_{2},j}^{\prime } & = & C_{\ell_{2}}^{0}+\sum_{S}C_{\ell_{2},S}^{1}\delta_{Sj},\;\;1\leq j\leq 161, \end{array}$$

where $C_{\ell_{k}}^{0}$ and $C_{\ell_{k},S}^{1}$ (*k* = 1, 2) are regression coefficients. *δ*_*Sj*_ takes 1 when *j*th sample belongs to category *S*, and isotherwise 0.

Then, assuming that PC scores *u*_*ℓ*_1_,*i*_1__s (for mRNA) and $u_{\ell_{2},i_{2}}^{\prime }\text{s}$ (for miRNA) are normally distributed, the *P*-values were attributed to the *i*_1_th mRNA and *i*_2_th miRNA using a *χ* squared distribution as

$$\begin{array}{ccc} P_{i_{1}} & = & P_{\chi^{2}}\left[>\sum_{\ell_{1}\leq 5}{\left(\frac{u_{\ell_{1},i_{1}}}{\sigma_{u_{\ell_{1}}}}\right)}^{2}\right]\\ P_{i_{2}} & = & P_{\chi^{2}}\left[>\sum_{\ell_{2}\leq 5}{\left(\frac{u_{\ell_{2},i_{2}}^{\prime }}{\sigma_{u_{\ell_{2}}^{\prime }}}\right)}^{2}\right], \end{array}$$

where *σ*_*u*_*ℓ*_1___ and $\sigma_{u_{\ell_{2}}^{\prime }}$ is the standard deviation of {*u*_*ℓ*_1_,*i*_1__|1 ≤ *i*_1_ ≤ 13393} and $\left\{u_{\ell_{2},i_{2}}^{\prime }|1\leq i_{2}\leq 755\right\}$, respectively. *P*_*χ*^2^_[>*x*] is the probability that the argument is larger than *x* under the assumption that the arguments obey a *χ* squared distribution. The *P*-values were further adjusted by the Benjamini-Hochberg (BH) criterion [26], and those genes associated with adjusted *P*-values less than 0.01 were selected as the genes associated with the difference between multiple classes.

#### PCA-based unsupervised FE applied to vaccination experiments

Although similar to the previous section, some differences are: (i) Only the second PC scores attributed to mRNAs were used for FE. (ii) Instead of mRNA and miRNA, patient category groups, P, D, and NP were used. (iii) mRNAs commonly included in three independent FEs performed considering P, D, and NP, were considered.

### TD-based unsupervised FE

For type I tensor generated from multi-omics dataset, in order to identify miRNAs and mRNAs associated with identified sample singular value vectors, it was assumed that $x_{\ell_{1},i_{1}}^{\text{mRNA}}$ and $x_{\ell_{2},i_{2}}^{\text{miRNA}}$ follow multiple normal distributions, and *P*-values were attributed to the *i*_1_th mRNA and the *i*_2_th miRNA using *χ*^2^ distribution.

$$\begin{array}{c} P_{i_{1}}=P_{\chi^{2}}\left[>\sum_{\ell_{1}\leq 5}{\left(\frac{x_{\ell_{1},i_{1}}^{\text{mRNA}}}{\sigma_{\ell_{1}}}\right)}^{2}\right],\;\;P_{i_{2}}=P_{\chi^{2}}\left[>\sum_{\ell_{k}\leq 2}{\left(\frac{x_{\ell_{2},i_{2}}^{\text{miRNA}}}{\sigma_{\ell_{2}}}\right)}^{2}\right] \end{array}$$

where *σ*_*ℓ*_1__(*σ*_*ℓ*_2__) are standard deviations of $x_{\ell_{1},i_{1}}^{\text{mRNA}}\left(x_{\ell_{2},i_{2}}^{\text{miRNA}}\right)$. *P*_*i*_*k*__s were adjusted by using the BH criterion, and mRNAs and miRNAs associated with the adjusted *P*-value lower than 0.01 for mRNA and 0.05 for miRNA were selected as those associated with identified sample singular value vectors when TD was applied to type I tensor.

When TD was applied to type II tensor, the computations were similar, excluding that adjusted *P*-value lower than 0.01 and 1 ≤ *ℓ*_2_ ≤ 5 were used for miRNA, where $x_{\ell_{1},i_{1}}^{\text{mRNA}}$ and $x_{\ell_{2},i_{2}}^{\text{miRNA}}$ were replaced with ${\tilde{x}}_{\ell_{1},i_{1}}^{\text{mRNA}}$ and ${\tilde{x}}_{\ell_{2},i_{2}}^{\text{miRNA}}$, respectively.

For EGF treated cell lines and vaccination experiments, similar procedures were repeated by replacing gene singular value vectors with *x*_*ℓ*_3_ = 2,*i*_ (EGF, type I) or ${\tilde{x}}_{\ell_{3}=2,i}^{\text{control}}$ and ${\tilde{x}}_{\ell_{3}=2,i}^{\text{EGF}}$ (EGF, type II) or ${\tilde{x}}_{\ell_{3}=2,i}^{\text{D}}$, ${\tilde{x}}_{\ell_{3}=2,i}^{\text{P}}$ and ${\tilde{x}}_{\ell_{3}=2,i}^{\text{NP}}$, (vaccination, type II), respectively.

Where HO GSVD was applied to multi-omics datasets, the feature singular value matrices were replaced with the first five column vectors of *U*^(*k*)^ (*k* = 1 for mRNA and *k* = 2 for miRNA).

Adjusted *P* values used as thresholds are always 0.01 for EGF treated cell lines, vaccination, and HO GSVD.

### Conversion prob ID to HUGO gene name/ensembl gene ID/GENBANK accession ID

Coincidences between prob ID (mRNA), HUGO gene names, Ensembl gene IDs were downloaded from GEO using GEO ID GPL3676 for multi-omics datasets, and GPL571/570 for vaccination experiments. GENBANK accession ID attributed to probes identified in the EGF treatment were extracted from GPL16686.

### Enrichment analysis of g:profiler

Ensembl gene IDs were uploaded to g:profiler [27]. 13393 Ensembl gene IDs were used as background.

### Enrichment analysis of genes identified as outliers using each of the first five mRNA singular value vectors obtained by applying TD based unsupervised FE to type I tensor generated from multi-omics datasets

Five distinct *P*-values were attributed to the *i*_1_th mRNA using *χ*^2^ distribution:

$$\begin{array}{c} P_{i_{1}}^{\ell_{1}}=P_{\chi^{2}}\left[>{\left(\frac{x_{\ell_{1},i_{1}}^{\text{mRNA}}}{\sigma_{\ell_{1}}}\right)}^{2}\right],\;1\leq \ell_{1}\leq 5 \end{array}$$

*P*-values were adjusted using the BH criterion and mRNAs associated with adjusted *P*-values less than 0.01 were identified as outliers (see S2 Table). S2 Table was uploaded to g:Cocoa in g:profiler with “Gene Ontology/ Biological Process” specified as the targeted ontology.

### Enrichment analysis of MSigDB

HUGO gene IDs or GENBANK accession IDs associated with identified mRNAs were uploaded to http://software.broadinstitute.org/gsea/msigdb/annotate.jsp (registration and login are needed). “CGP: chemical and genetic perturbations” was selected for multi-omics data and EGF treated cell lines, while “C7: immunologic signatures” was selected for vaccination experiments.

### Enrichment analysis of DIANA-mirpath

As for TDs applied to type I tensors, the following link was pasted to browser.

http://snf-515788.vm.okeanos.grnet.gr/#mirnas=hsa-let-7b-5p;hsa-miR-125b-5p;hsa-miR-143-3p;hsa-miR-145-5p;hsa-miR-21-5p;hsa-miR-22-3p;hsa-miR-99a-5p&methods=Tarbase;Tarbase;Tarbase;Tarbase;Tarbase;Tarbase;Tarbase&selection=0

As for TDs applied to type II tensors, the following link was pasted to browser.

http://snf-515788.vm.okeanos.grnet.gr/#mirnas=hsa-let-7a-5p;hsa-let-7b-5p;hsa-let-7f-5p;hsa-miR-103a-3p;hsa-miR-125b-5p;hsa-miR-141-3p;hsa-miR-142-3p;hsa-miR-143-3p;hsa-miR-145-5p;hsa-miR-148a-3p;hsa-miR-199a-3p;hsa-miR-199b-3p;hsa-miR-19b-3p;hsa-miR-205-5p;hsa-miR-21-5p;hsa-miR-22-3p;hsa-miR-23a-3p;hsa-miR-24-3p;hsa-miR-26a-5p;hsa-miR-30a-5p;hsa-miR-451a;hsa-miR-99a-5p&methods=Tarbase;Tarbase;Tarbase;Tarbase;Tarbase;Tarbase;Tarbase;Tarbase;Tarbase;Tarbase;Tarbase;Tarbase;Tarbase;Tarbase;Tarbase;Tarbase;Tarbase;Tarbase;Tarbase;Tarbase;Tarbase;Tarbase&selection=0

As for HO GSVD, the following link was pasted to browser.

http://snf-515788.vm.okeanos.grnet.gr/#mirnas=hsa-miR-127-5p;hsa-miR-128-1-5p;hsa-miR-181a-3p;hsa-miR-190a-5p;hsa-miR-301a-3p;hsa-miR-30e-3p;hsa-miR-339-5p;hsa-miR-340-5p;hsa-miR-361-5p;hsa-miR-365a-3p;hsa-miR-452-5p;hsa-miR-454-3p;hsa-miR-455-5p;hsa-miR-874-5p;hsa-miR-135a-5p&methods=Tarbase;Tarbase;Tarbase;Tarbase;Tarbase;Tarbase;Tarbase;Tarbase;Tarbase;Tarbase;Tarbase;Tarbase;Tarbase;Tarbase;Tarbase&selection=0

### Categorical regression towards data shown in Fig 1

**Fig 1:**
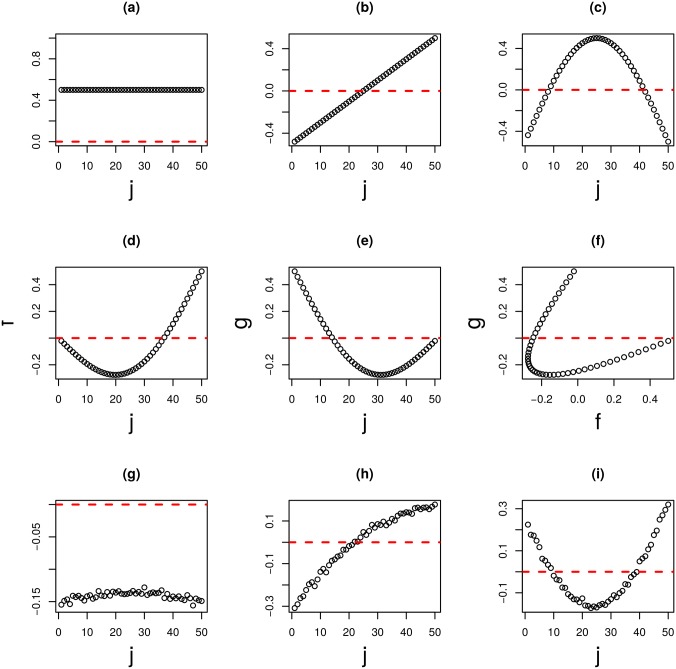
Boxplots of sample singular value vectors *x*_*ℓ*_3_,*j*_ (a) when TD was applied to the type I tensor and ${\tilde{x}}_{\ell_{3},j}^{\text{mRNA}}$ (b), ${\tilde{x}}_{\ell_{3},j}^{\text{miRNA}}$ (c), 1 ≤ *ℓ*_3_ ≤ 5, when TD was applied to the type II tensor, generated from mRNA and miRNA expression profiles of multi-omics datasets. (d) Sample singular value vectors when HO GSVD was applied to multi-omics datasets. *P*-values computed by categorical regression attributed to (a) to (d) were below the figures.

For type I tensor (Fig 1(a)),

$$\begin{array}{ccc} x_{\ell_{3},j} & = & C_{\ell_{3}}^{0}+\sum_{S}C_{\ell_{3},S}^{1}\delta_{Sj},\;\;1\leq j\leq 161 \end{array}$$

where $C_{\ell_{3}}^{0}$ and $C_{\ell_{3},S}^{1}$ are regression coefficients. *δ*_*Sj*_ takes 1 when *j*th sample belongs to category *S*, otherwise 0. The summation is taken over all categories. For type II tensor (Fig 1(b) and 1(c)), *x*_*ℓ*_3_,*j*_ is replaced with ${\tilde{x}}_{\ell_{3},j}^{\text{mRNA}}$ or ${\tilde{x}}_{\ell_{3},j}^{\text{miRNA}}$. For HO GSVD (Fig 1(d)), *x*_*ℓ*_3_,*j*_ is replaced with column vectors of *V*.

### Correlations in Fig 2(c) and 2(g)

**Fig 2:**
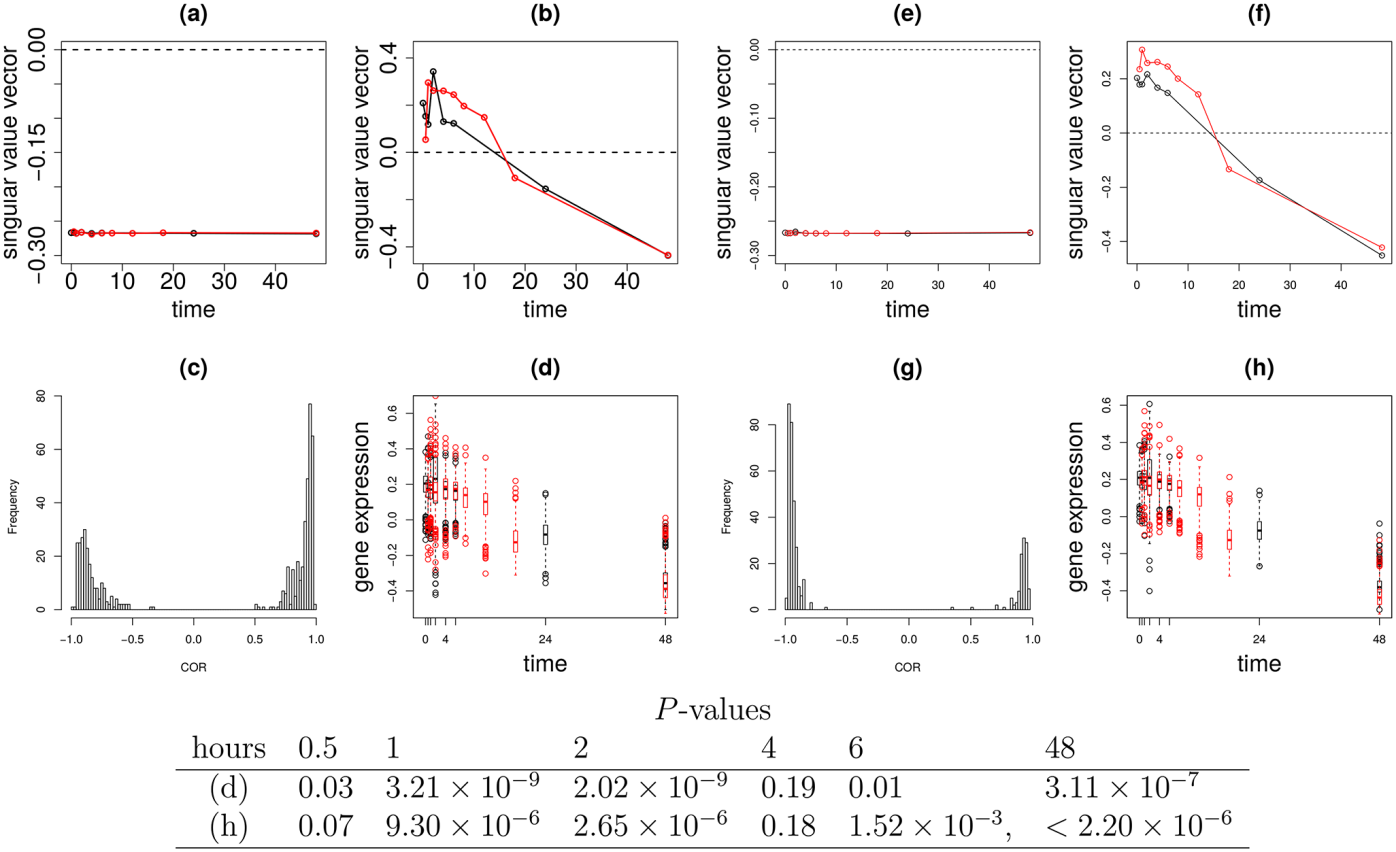
The results of TD applied to type I tensor generated from EGF treatment experiments. Sample singular value vectors, Black open circle: $x_{\ell_{1},t_{1}}^{\text{control}}$ Red open circle: $x_{\ell_{2},t_{2}}^{\text{EGF}}$ (a) *ℓ*_1_ = 1 (b) *ℓ*_1_ = 2. (c) Histogram of the correlation coefficients between sample (time) singular value vectors and selected individual 558 mRNA probes expression profiles. (d) Boxplot of scaled and shifted selected individual 558 mRNA probe expression profiles. Black: control, Red: EGF treated cell lines. The same as (a) to (d), but for type II tensor. Black open circles: ${\tilde{x}}_{\ell_{1},t_{1}}^{\text{control}}$ Red open circles: ${\tilde{x}}_{\ell_{2},t_{2}}^{\text{EGF}}$ (e) *ℓ*_1_ = 1 (f) *ℓ*_1_ = 2. (g) Histogram of the correlation coefficients between sample (time) singular value vectors and selected individual 398 mRNA probe expression profiles. (h) Boxplot of scaled and shifted selected individual 398 mRNA probe expression profiles. Black: control, Red: EGF treated cell lines. *P*-values computed by *t* test of 558 (d) or 398 (h) mRNA probes between with and without EGF treatments are below figures.

The correlations were computed between two vectors of the length *T*_control_ + *T*_EGF_, $\left(x_{\ell_{1}=2,t_{1}=1}^{\text{control}},\ldots ,x_{\ell_{1}=2,t_{1}=T_{\text{control}}}^{\text{control}},x_{\ell_{2}=2t_{2}=1}^{\text{EGF}},\ldots ,x_{\ell_{2}=2,t_{2}=T_{\text{EGF}}}^{\text{EGF}}\right)$ and $\left(x_{i,t_{1}=1}^{\text{control}},\ldots ,x_{i,t_{1}=T_{\text{control}}}^{\text{control}},x_{i,t_{2}=1}^{\text{EGF}},\ldots ,x_{i,t_{2}=T_{\text{EGF}}}^{\text{EGF}}\right)$ (Fig 2(c)) or between $\left({\tilde{x}}_{\ell_{1}=2,t_{1}=1}^{\text{control}},\ldots ,{\tilde{x}}_{\ell_{1}=2,t_{1}=T_{\text{control}}}^{\text{control}},{\tilde{x}}_{\ell_{2}=2,t_{2}=1}^{\text{EGF}},\ldots ,{\tilde{x}}_{\ell_{2}=2,t_{2}=T_{\text{EGF}}}^{\text{EGF}}\right)$ and $\left(x_{i,t_{1}=1}^{\text{control}},\ldots ,x_{i,t_{1}=T_{\text{control}}}^{\text{control}},x_{i,t_{2}=1}^{\text{EGF}},\ldots ,x_{i,t_{2}=T_{\text{EGF}}}^{\text{EGF}}\right)$ (Fig 2(g)), where *T*_control_ and *T*_EGF_ are total number of samples in each treatment, respectively. Adjusted *P*-values attributed to the correlation coefficients were computed via the fdrtool [28] function in the fdrtool package in R [29].

### Scaling and shifting prior to plotting Fig 2(d) and 2(h)

As each individual gene expression has its own base line and amplitude, they must be scaled and shifted before being overdrawn. To this end, the linear regression analysis

$$\begin{array}{ccc} x_{\ell_{1}=2,t_{1}}^{\text{control}} & = & a_{i}x_{i,t_{1}}^{\text{control}}+b_{i},\;t_{1}=1,\ldots ,T_{\text{control}}\\ x_{\ell_{2}=2,t_{2}}^{\text{EGF}} & = & a_{i}x_{i,t_{2}}^{\text{EGF}}+b_{i},\;t_{2}=1,\ldots ,T_{\text{EGF}} \end{array}$$

was employed (Fig 2(c)) where *a*_*i*_ and *b*_*i*_ are regression coefficients commonly used for control and EGF treated samples. For Fig 2(g), $x_{\ell_{1}=2,t_{1}}^{\text{control}}$ and $x_{\ell_{2}=2,t_{2}}^{\text{EGF}}$ are replaced with ${\tilde{x}}_{\ell_{1}=2,t_{1}}^{\text{control}}$ and ${\tilde{x}}_{\ell_{2}=2,t_{2}}^{\text{EGF}}$, respectively. Then, fitted values are used for plots. *P*-values that exhibit distinction between control and EGF treated sample at time point *t* were computed by two-sided *t* test between $\left\{a_{i}x_{i,t_{1}=t}^{\text{control}}+b_{i}|1\leq i\leq N\right\}$ and $\left\{a_{i}x_{i,t_{2}=t}^{\text{EGF}}+b_{i}|1\leq i\leq N\right\}$ within *N* = 558 (Fig 2(d)) or *N* = 398 (Fig 2(h)) selected mRNA probes.

### Correlations in Fig 3(c)

Similar to Fig 2, the correlations were computed between two vectors of length *T*_P_ + *T*_D_ + *T*_NP_, $\left({\tilde{x}}_{\ell_{4}=2,t_{1}=1}^{\text{P}},\ldots ,{\tilde{x}}_{\ell_{4}=2,t_{1}=T_{\text{P}}}^{\text{P}},{\tilde{x}}_{\ell_{4}=2,t_{2}=1}^{\text{D}},\ldots ,{\tilde{x}}_{\ell_{4}=2,t_{2}=T_{\text{D}}}^{\text{D}},x_{\ell_{4}=2,t_{3}=1}^{\text{NP}},\ldots ,x_{\ell_{4}=2,t_{3}=T_{\text{NP}}}^{\text{NP}}\right)$ and $(x_{i,t_{1}=1}^{\text{P}},\ldots ,x_{i,t_{1}=T_{\text{P}}}^{\text{P}},x_{i,t_{2}=1}^{\text{D}},\ldots ,x_{i,t_{2}=T_{\text{D}}}^{\text{D}},x_{i,t_{3}=1}^{\text{NP}},\ldots ,{\tilde{x}}_{i2,t_{3}=T_{\text{NP}}}^{\text{NP}}),$ where *T*_P_ = 58, *T*_D_ = 52, and *T*_NP_ = 72 are the total number of samples in each patient category, respectively. Adjusted *P*-values were computed via the fdrtool function in the fdrtool package in R [29].

**Fig 3:**
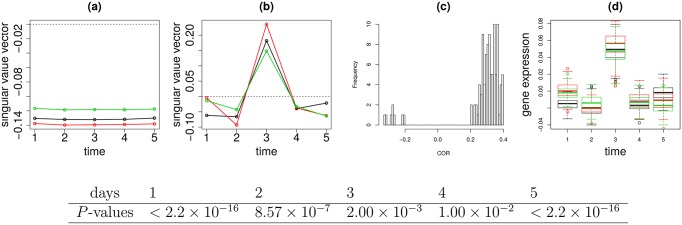
The results of TD applied to type II tensor generated from vaccination. Sample singular value vectors, Black open circle: ${\tilde{x}}_{\ell_{1},t_{1}}^{\text{P}}$ Red open circle: ${\tilde{x}}_{\ell_{2},t_{2}}^{\text{D}}$ Green open circle: ${\tilde{x}}_{\ell_{3},t_{3}}^{\text{NP}}$ (a) *ℓ*_1_ = *ℓ*_2_ = *ℓ*_3_ = 1 (b) *ℓ*_1_ = *ℓ*_2_ = *ℓ*_3_ = 2 (c) Histogram of the correlation coefficients between sample singular value vectors and selected individual 104 mRNA probes expression profiles. (d) Boxplot of scaled and shifted selected individual 104 mRNA probe expression profiles. Black: P, Red:D, green:ND cell lines. *P*-values computed by categorical regression between P, D, and NP groups are below figures.

### Scaling and shifting prior to plotting Fig 3(d)

The linear regression analysis

$$\begin{array}{ccc} {\tilde{x}}_{\ell_{1}=2,t_{1}}^{\text{P}} & = & a_{i}x_{i,t_{1}}^{\text{P}}+b_{i},\;t_{1}=1,\ldots ,T_{\text{P}}\\ {\tilde{x}}_{\ell_{2}=2,t_{2}}^{\text{D}} & = & a_{i}x_{i,t_{2}}^{\text{D}}+b_{i},\;t_{2}=1,\ldots ,T_{\text{D}}\\ {\tilde{x}}_{\ell_{3}=2,t_{3}}^{\text{NP}} & = & a_{i}x_{i,t_{3}}^{\text{NP}}+b_{i},\;t_{3}=1,\ldots ,T_{\text{NP}} \end{array}$$

was employed, where *a*_*i*_ and *b*_*i*_ are regression coefficients commonly used for three patient categories (these values differ from those used in Fig 2). Fitted values are used for plots. *P*-values that exhibit distinction among three patient groups at time point *t* = 1, 2, 3, 4, 5 days were computed by categorical regression

$$\begin{array}{c} a_{i}x_{i,t}^{S}+b_{i}=C_{t}^{0}+\sum_{S^{\prime }\in \left(\text{P,D,NP}\right)}C_{tS^{\prime }}^{1}\delta_{SS^{\prime }} \end{array}$$

for 104 commonly selected mRNA probes in the three categories. $C_{t}^{0}$ and $C_{tS^{\prime }}^{1}$ are regression coefficients fitted for $\left\{x_{i,t}^{S}|1\leq i\leq 104,S\in \left(\text{P},\text{D},\text{NP}\right)\right\}$ with fixed *t*.

### Statistical analysis

All statistical analyses were performed in R [29]. HOSVD was performed using the HOSVD function in the rTensor package. PCA was performed using the prcomp function in R. SAM was performed using SAM function in siggenes package. limma was performed in limma function in the limma package. Adjusted *P*-values were computed by p.adjust function with “BH” options. *P*-values by *χ*^2^ distribution was computed by pchisq function in R. Categorical regression was performed using the lm function in R. RF was performed using randomForest function in randomForest package. KCCA was performed by KCCA function in the kernlab package.

## Results

A Work flow chart and list of the variables introduced are in S1 File.

### Synthetic dataset

In order to demonstrate the efficacy of our strategy, I applied TD based unsupervised FE to synthetic data. In the following interpretation, I assumed two views of Case I for synthetic dataset, however interpreting it as Case II is straightforward, thus, I do not consider Case II specifically. The method applied to the synthetic dataset, TD based unsupervised FE, is the extension from the recently proposed PCA based unsupervised FE, which has been successfully applied to various bioinformatics problems [5–22].

**Fig 4:**
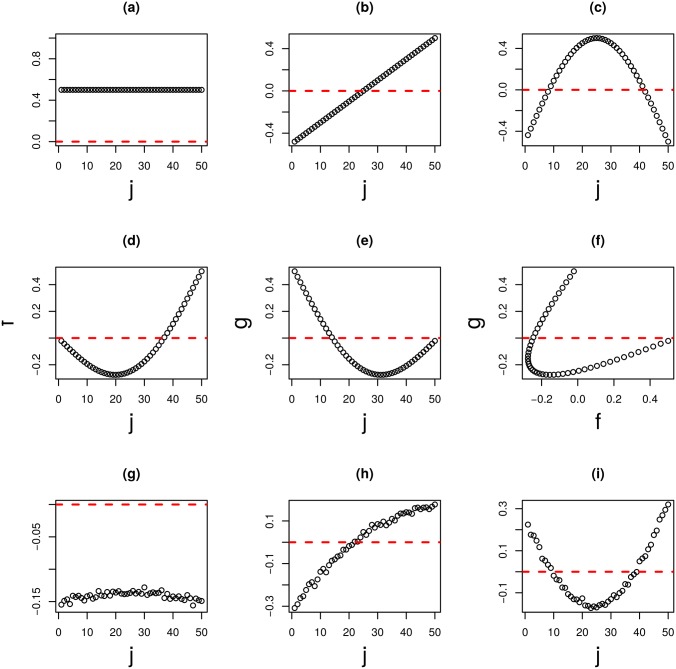
The results of TD applied to the type I tensor generated from a synthetic dataset (*M* = 50). (a) to (c) are orthogonal base functions: (a) constant, (b) linear, (c) half period sinusoidal. (d) and (e) base functions used for generating $x_{i_{k},j}^{\left(k\right)},1\leq i_{k}\leq N_{0}$. (d) *k* = 1, (e) *k* = 2. (f) is the scatter plot of (d) and (e). (g) to (i) are the first, second, and third sample singular value vectors *x*_*ℓ*_3_,*j*_ and *ℓ*_3_ = 1, 2, 3, and are computed by applying TD to synthetic data.

First, two matrices are generated, each of which is composed of *N* features times *M* samples. They are notated as *X*^(*k*)^, and *k* = 1, 2, respectively, whose components are denoted as $x_{i_{k},j}^{\left(k\right)}$, and *k* = 1, 2, respectively. The first *N*_0_ row vectors (features), $x_{i_{k},j}^{\left(k\right)}$, and 1 ≤ *i*_*k*_ ≤ *N*_0_, are the noise added linear combination (Fig 4(d) and 4(e)) of constant, linear, and half period sinusoidal function (Fig 4(a) and 4(c)). However, since the coefficients of linear combinations were selected such that the correlation between *X*^(1)^ and *X*^(2)^ is negligible, identifying a correlation between two matrix row vectors is usually impossible (Fig 4(f)). Remaining row vectors $x_{i_{k},j}^{\left(k\right)}$ and *N*_0_ < *i*_*k*_ ≤ *N* are simply random number ∈ [0, 1]. The tasks are (i) identify *N*_0_ ordered features, and (ii) identify latent correspondence between two views.

The three mode tensor (type I) $x_{i_{1},i_{2},j}=x_{i_{1},j}^{\left(1\right)}x_{i_{2},j}^{\left(2\right)},1\leq i_{1},i_{2}\leq N,1\leq j\leq M$ was derived from $x_{i_{k},j}^{\left(k\right)},k=1,2$. Fig 4(g)–4(i) shows the first three sample mode singular value vectors, *x*_*ℓ*_3_,*j*_, *ℓ*_3_ = 1, 2, 3, obtained with HOSVD,

$$\begin{array}{c} x_{i_{1},i_{2},j}=x_{i_{1},j}^{\left(1\right)}x_{i_{2},j}^{\left(2\right)}=\sum_{\ell_{1}=1}^{N}\sum_{\ell_{2}=1}^{N}\sum_{\ell_{3}=1}^{M}G\left(\ell_{1},\ell_{2},\ell_{3}\right)x_{\ell_{1},i_{1}}^{\left(1\right)}x_{\ell_{2},i_{2}}^{\left(2\right)}x_{\ell_{3},j} \end{array}$$ (3)

It is obvious that Fig 4(g)–4(i) corresponds to Fig 4(a)–4(c), respectively. To my knowledge, it is the only method to decompose linear combinations back into parts in a fully unsupervised manner.

**Fig 5:**
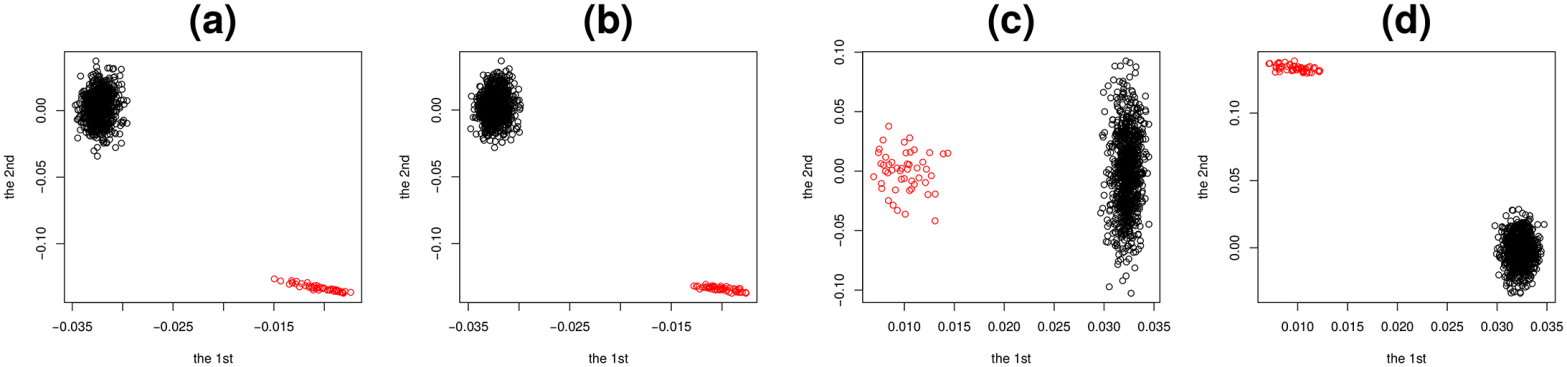
Feature singular value vectors when TD was applied to type I tensor generated from synthetic data. (a) $x_{\ell_{1},i_{1}}^{\left(1\right)},\ell_{1}=1,2$ and (b) $x_{\ell_{2},i_{2}}^{\left(2\right)},\ell_{2}=1,2$. and type II tensor, (c) ${\tilde{x}}_{\ell_{1},i_{1}}^{\left(1\right)},\ell_{1}=1,2$ and (d) ${\tilde{x}}_{\ell_{2},i_{2}}^{\left(2\right)},\ell_{2}=1,2$. Red open circles are 1 ≤ *i*_1_, *i*_2_ ≤ *N*_0_ and black open circles are *N*_0_ < *i*_1_, *i*_2_ ≤ *N*.

Moreover, as can be seen in Fig 5(a) and 5(b), *N*_0_ features are placed as outliers. Thus, TD based unsupervised FE applied to type I tensors generated from matrices’ products can not only decompose linear combinations, but can also identify the limited number of features, 1 ≤ *i*_1_, *i*_2_, ≤ *N*_0_, that contribute to correlations between two matrices, *X*^(*k*)^, *k* = 1, 2.

Although I could successfully demonstrate that my strategy works well, there is one drawback; it is the computationally extensive method, since its memory as well as computational time are proportional to *MN*^2^. In order to reduce computational resources as much as possible, I summed $x_{i_{1},j}^{\left(1\right)}x_{i_{2},j}^{\left(2\right)}$ and generated the *m*(= 2) mode tensor (type II), ${\tilde{x}}_{i_{1},i_{2}}=\sum_{j}x_{i_{1},j}^{\left(1\right)}x_{i_{2},j}^{\left(2\right)}$. Then, ${\tilde{x}}_{i_{1},i_{2}}$ is decomposed as

$$\begin{array}{ccc} {\tilde{x}}_{i_{1},i_{2}} & = & \sum_{j}x_{i_{1},j}^{\left(1\right)}x_{i_{2},j}^{\left(2\right)}=\sum_{\ell_{1}}^{N}\sum_{\ell_{2}}^{N}\tilde{G}\left(\ell_{1},\ell_{2}\right){\tilde{x}}_{\ell_{1},i_{1}}^{\left(1\right)}{\tilde{x}}_{\ell_{2},i_{2}}^{\left(2\right)} \end{array}$$ (4)

Two sample singular value vectors can be computed as

$$\begin{array}{c} {\tilde{x}}_{\ell_{3}=\ell_{k},j}^{\left(k\right)}=\sum_{i_{k}}{\tilde{x}}_{\ell_{k},i_{k}}^{\left(k\right)}x_{i_{k},j}^{\left(k\right)},\;k=1,2. \end{array}$$

Since I employed the two views problem, although I occasionally got the two mode tensor, i.e., the matrix, if I consider *m*(>2) views, I generally get a *m* mode tensor and the above procedures can be easily extended to *m* mode tensors.

**Fig 6:**
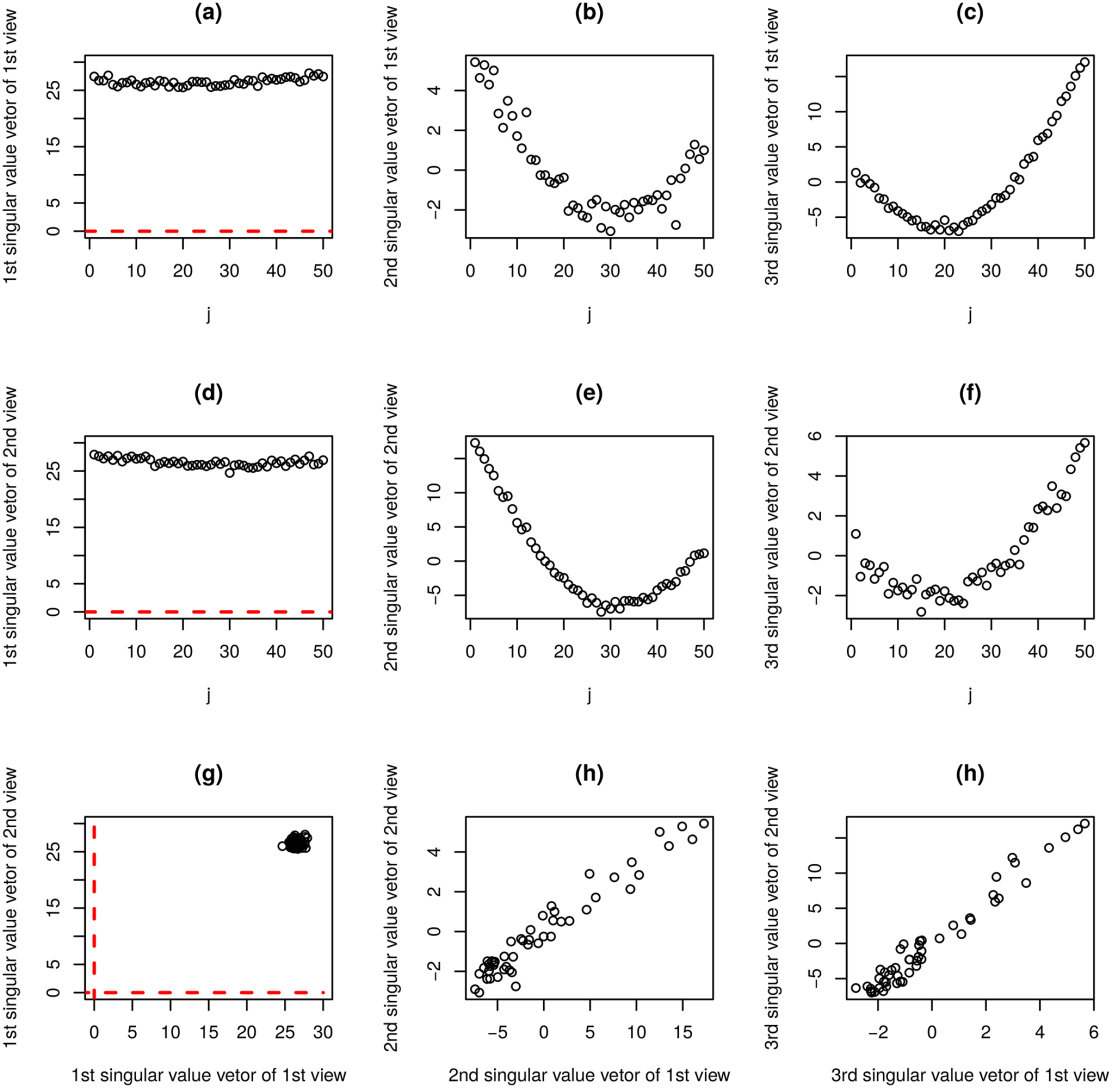
The results of TD applied to type II tensor generated from synthetic dataset (*M* = 50). ${\tilde{x}}_{\ell_{3},j}^{\left(1\right)}$ (a) *ℓ*_3_ = 1 (b) *ℓ*_3_ = 2 (c) *ℓ*_3_ = 3. ${\tilde{x}}_{\ell_{3},j}^{\left(2\right)}$ (d) *ℓ*_3_ = 1 (e) *ℓ*_3_ = 2 (f) *ℓ*_3_ = 3. (g): (a) vs (c), (h): (b) vs (d), *γ* = 0.97, *P* = 0, (i): (c) vs (f), *γ* = 0.97, *P* = 0. *γ*: Pearson correlation coefficients. P: associated *P*-values.

Fig 6 shows the first three sample singular value vectors, ${\tilde{x}}_{\ell_{3},j}^{\left(k\right)},\ell_{3}=1,2,3$, and (*k* = 1, 2), obtained with the application of HOSVD to type II tensors. Among these, ${\tilde{x}}_{\ell_{3},j}^{\left(k\right)}$ and *k* = 1, 2, shown in Fig 6(b) and 6(e) (*ℓ*_3_ = 1) correctly reproduce Fig 4(e) while those shown in Fig 6(c) and 6(f) (*ℓ*_3_ = 2) does Fig 4(d), respectively. Thus, TD applied to type II tensors also successfully identified latent correlations between *X*^(*k*)^, *k* = 1, 2 (Fig 6(h) and 6(i)). However, it could not depict orthogonal base functions (Fig 4(a)–4(c)) that can be detected by TD applied to type I tensors (Fig 4(g)–4(i)). Additionally, *N*_0_ features were successfully identified as outliers (Fig 5(c) and 5(d)). Thus, at the expense of recognition of orthogonal base functions, TD applied to type II tensors successfully reduced the computational resources needed by 1/*M* and fulfilled tasks (i) and (ii) as defined above.

In order to see if these strategies are also useful in practice, integrated analyses of multi-omics datasets were performed with this strategy and are described in the next subsection.

### Multi-omics dataset

In the previous subsection, I demonstrated that applying TD based unsupervised FE to tensors generated from matrices’ products could determine latent structures behind pairs of matrices. However, this may only be feasible using synthetic data, as described in the previous subsection. Thus, in order to see if it also works in the situation not prepared specifically fitted to it, I need to show that it works in real situation.

The analysed dataset is composed of two omics profiles. These are mRNA and miRNA profiles which were measured for multi-class breast cancer samples including normal breast tissues [30]. As the samples are shared, the multi-omics data corresponds to Case I data. TD based unsupervised FE was applied to the dataset in order to identify disease critical genes and latent relations between miRNA and mRNA.

At first, TD was applied to the type I tensor generated from the mRNA and miRNA profiles as follows:

$$\begin{array}{ccc} x_{i_{1},i_{2},j} & = & x_{i_{1},j}^{\text{mRNA}}x_{i_{2},j}^{\text{miRNA}}\\ & = & \sum_{\ell_{1}}\sum_{\ell_{2}}\sum_{\ell_{3}}G\left(\ell_{1},\ell_{2},\ell_{3}\right)x_{\ell_{1},i_{1}}^{\text{mRNA}}x_{\ell_{2},i_{2}}^{\text{miRNA}}x_{\ell_{3},j} \end{array}$$

where $x_{i_{1},j}^{\text{mRNA}}$ and $x_{i_{2},j}^{\text{miRNA}}$ are expressions of the *i*_1_th mRNA and the *i*_2_th miRNA from the *j*th sample. In order to determine whether TD can identify disease critical features, categorical regression analysis was applied to sample singular value vector *x*_*ℓ*_3_,*j*_, in order to identify coincidences with defined sample classes. If the obtained sample singular value vectors are coincident with sample class labels, it is evidence that TD can process omics profiles properly, as this approach does not employ class labels explicitly. Fig 1 shows the first five sample singular value vectors, *x*_*ℓ*_3_,*j*_, 1 ≤ *ℓ*_3_ ≤ 5, that show significant sample class dependence. Thus, TD could successfully generate disease associated features. This is not a trivial outcome, as sample classification was not used.

Next, I attempted to extract features using the mRNA and miRNA singular value vectors $x_{\ell_{1},i_{1}}^{\text{mRNA}}$ and $x_{\ell_{2},i_{2}}^{\text{miRNA}}$. To accomplish this, it was necessary to first identify mRNA and miRNA singular value vectors $x_{\ell_{1},i_{1}}^{\text{mRNA}}$ and $x_{\ell_{2},i_{2}}^{\text{miRNA}}$, associated with sample singular value vectors, *x*_*ℓ*_3_,*j*_, 1 ≤ *ℓ*_3_ ≤ 5 identified above. This can be done by investigating *G*(*ℓ*_1_, *ℓ*_2_, *ℓ*_3_) since the combinations (*ℓ*_1_, *ℓ*_2_, *ℓ*_3_) associated with larger absolute *G* values are regarded as more coincident with one another. Table 1 shows the ranking of *G* for 1 ≤ *ℓ*_1_, *ℓ*_2_, *ℓ*_3_ ≤ 5 (ranked from 1 to 10) based upon their absolute values. The first five sample singular value vectors *x*_*ℓ*_3_,*j*_, 1 ≤ *ℓ*_3_ ≤ 5, are associated with the first two miRNA singular value vectors $x_{\ell_{2},i_{2}}^{\text{miRNA}},\ell_{2}=1,2$ as well as the first five mRNA singular value vectors, $x_{\ell_{1},i_{1}}^{\text{mRNA}},1\leq \ell_{1}\leq 5$, as only these combinations appear within top ten ranked *G*(*ℓ*_1_, *ℓ*_2_, *ℓ*_3_)s that represent the amount of coincidence among mRNA and miRNA and samples.

**Table 1:** Top ranked 10 *G*(*ℓ*_1_, *ℓ*_2_, *ℓ*_3_)s with larger absolute values among 1 ≤ *ℓ*_1_, *ℓ*_2_, *ℓ*_3_ ≤ 10 when TD was applied to type I tensor generated from $x_{i_{1},j}^{\text{mRNA}}$ and $x_{i_{2},j}^{\text{miRNA}}$ (left) and $x_{i,t_{1}}^{\text{control}}$ and $x_{i,t_{2}}^{\text{EGF}}$ (right).

multi-omics				EGF treatment			
*ℓ*_1_	*ℓ*_2_	*ℓ*_3_	*G*(*ℓ*_1_, *ℓ*_2_, *ℓ*_3_)	*ℓ*_1_	*ℓ*_2_	*ℓ*_3_	*G*(*ℓ*_1_, *ℓ*_2_, *ℓ*_3_)
1	1	1	1.67 × 10^5^	1	1	1	−4.03 × 10^4^
2	1	2	−1.03 × 10^5^	2	1	2	−1.56 × 10^3^
4	1	4	7.48 × 10^4^	1	2	2	1.49 × 10^3^
3	1	3	−6.64 × 10^4^	3	1	3	1.05 × 10^3^
5	1	5	6.23 × 10^4^	1	3	4	−5.79 × 10^2^
3	2	3	3.00 × 10^4^	4	1	5	4.24 × 10^2^
1	2	3	−2.87 × 10^4^	2	1	3	4.16 × 10^2^
3	1	5	−2.33 × 10^4^	5	1	6	3.25 × 10^2^
2	2	3	−2.02 × 10^4^	1	4	6	3.19 × 10^2^
1	2	2	−1.48 × 10^4^	4	1	4	−2.62 × 10^2^

Four hundred twenty-seven mRNA probes and 7 miRNAs, identified as outliers, using $x_{\ell_{1},i_{1}}^{\text{mRNA}},1\leq \ell_{1}\leq 5$ and $x_{\ell_{2},i_{2}}^{\text{miRNA}},1\leq \ell_{2}\leq 2$ were selected. The seven selected miRNAs were: hsa-let-7b, hsa-miR-125b, hsa-miR-143, hsa-miR-145, hsa-miR-21, hsa-miR-22, hsa-miR-99a. Remarkably, 143, 145, 21, 22, 99a as well as let-7a, and 125a, which belongs to the same families as 125b and let-7b, were also reported by the original study ([30], Table 1). The mRNAs associated with selected 427 mRNA probes are in S1 Table because of too many numbers.

In order to evaluate obtained mRNAs associated with the 427 selected mRNA probes and 7 miRNAs biologically, the mRNAs were uploaded for enrichment analysis using MSigDB [31] and the miRNAs to DIANA-mirpath [32]. The top 10 enriched gene sets in MSigDB are chiefly related to breast cancer (eight out of ten, see Table 2), while the top ranked pathways identified DIANA-mirpath are “MicroRNAs in cancer” (Table 3). Thus, I concluded that our strategy is successful despite fully unsupervised nature.

**Table 2:** Overlap between mRNAs identified (S1 Table) and MSigDB. Top 10 ranked gene sets are presented. Upper rows: type I, lower rows: type II tensors are considered in each gene set name, respectively. The word “BREAST_CANCER/_DUCTAL_CARCINOMA” was presented in bold face in order to emphasize the overlap with breast cancer related gene sets. K: The number of genes in each gene set, k: The number of genes overlapped.

Gene Set Name	(K)	Description	(k)	k/K	p-value	FDR q-value
SMID_**BREAST_CANCER**_LUMINAL_B_DN	564	Genes down-regulated in the luminal B subtype of breast cancer.	100	0.1773	4.00E-105	1.36E-101
			88	0.1560	2.34E-090	7.94E-087
SMID_**BREAST_CANCER**_BASAL_DN	701	Genes down-regulated in basal subtype of breast cancer samples.	91	0.1298	2.06E-082	3.50E-079
			86	0.1227	6.42E-079	1.09E-075
DOANE_**BREAST_CANCER**_ESR1_UP	112	Genes up-regulated in breast cancer samples positive for ESR1 compared to the ESR1 negative tumors.	44	0.3929	5.78E-063	6.56E-060
			38	0.3393	6.29E-053	4.28E-050
SMID_**BREAST_CANCER**_RELAPSE_IN_BONE_DN	315	Genes down-regulated in bone relapse of breast cancer.	—	—	—	—
			51	0.1619	1.17E-052	6.63E-050
JAEGER_METASTASIS_DN	258	Genes down-regulated in metastases from malignant tumors. melanoma compared to the primar	—	—	—	—
			43	0.1667	5.25E-045	2.55E-042
WALLACE_PROSTATE_CANCER_RACE_UP	299	Genes up-regulated in prostate cancer samples from African-American patients compared to those from the European-American patients.	55	0.1839	5.86E-058	4.98E-055
			—	—	—	—
SMID_**BREAST_CANCER**_NORMAL_LIKE_UP	476	Genes up-regulated in the normal-like subtype of breast cancer.	61	0.1282	2.40E-054	1.63E-051
			—	—	—	—
FARMER_**BREAST_CANCER**_BASAL_VS_LULMINAL	330	Genes which best discriminated between two groups of breast cancer according to the status of ESR1 and AR: basal (ESR1- AR-) and luminal (ESR1+ AR+).	54	0.1636	5.31E-054	3.01E-051
			54	0.1636	5.03E-056	4.27E-053
POOLA_INVASIVE_**BREAST_CANCER**_UP	288	Genes up-regulated in atypical ductal hyperplastic tissues from patients with (ADHC) breast cancer vs those without the cancer (ADH).	51	0.1771	7.55E-053	3.67E-050
			—	—	—	—
MCLACHLAN_DENTAL_CARIES_UP	254	Genes up-regulated in pulpal tissue extracted from carious teeth.	47	0.1850	1.12E-049	4.77E-047
			—	—	—	—
SMID_**BREAST_CANCER**_BASAL_UP	648	Genes up-regulated in basal subtype of breast cancer samples.	63	0.0972	1.38E-048	5.23E-046
			78	0.1204	1.18E-070	1.34E-067
SMID_**BREAST_CANCER**_LUMINAL_B_UP	172	Genes up-regulated in the luminal B subtype of breast cancer.	38	0.2209	1.95E-043	6.63E-041
			37	0.2151	3.29E-043	1.40E-040
DELYS_THYROID_UP_CANCER	443	Genes up-regulated in papillary thyroid carcinoma (PTC) compared to normal tissue.	—	—	—	—
			48	0.1084	5.47E-041	2.07E-038
TURASHVILI_**BREAST_DUCTAL_CARCINOMA**_VS_DUCTAL_NORMAL_DN	198	Genes down-regulated in ductal carcinoma vs normal ductal breast cells.	—	—	—	—
			36	0.1818	3.16E-039	1.08E-036

**Table 3:** Results of DIANA-mirath using seven miRNAs identified. Top 10 significant KEGG pathway was presented. gene: number of genes overlapped with miRNAs target genes, miRNA: number of overlapped miRNAs. Numbers both sides of “/” correspond to type I/type II tensors, respectively.

KEGG pathway	FDR q-value	gene	miRNA
MicroRNAs in cancer	3.29E-88/4.68E-68	115/141	7/22
Proteoglycans in cancer	5.36E-12/9.48E-17	116/159	7/22
Cell cycle	1.26E-10/2.61E-12	80/104	7/22
Renal cell carcinoma	—/2.25E-011	—/61	—/22
Protein processing in endoplasmic reticulum	—/3.81E-10	—/134	—/22
Hepatitis B	6.57E-09/5.37E-09	79/107	7/22
Prion diseases	5.14E-08/—	16/—	7/—
Central carbon metabolism in cancer	2.76E-07/—	42/—	7/—
Hippo signaling pathway	3.27E-07/2.40E-07	78/109	7/22
Chronic myeloid leukemia	—/2.40E-07	—/62	—/22
Viral carcinogenesis	—/2.40E-07	—/158	—/22
Pancreatic cancer	—/1.84E-06	—/55	—/22
Lysine degradation	1.15E-06/—	27/—	6/—
FoxO signaling pathway	2.89E-06/—	79/—	7/—
Prostate cancer	4.52E-06/—	56/—	7/—

Next, type II tensor generated from mRNA and miRNA profiles was considered. Applying TD to type II tensor,

$$\begin{array}{c} {\tilde{x}}_{i_{1},i_{2}}=\sum_{j}x_{i_{1},i_{2},j}=\sum_{\ell_{1}}\sum_{\ell_{2}}\tilde{G}\left(\ell_{1},\ell_{2}\right){\tilde{x}}_{\ell_{1},i_{1}}^{\text{mRNA}}{\tilde{x}}_{\ell_{2},i_{2}}^{\text{miRNA}}, \end{array}$$

gives us two sample singular value vectors

$$\begin{array}{ccc} {\tilde{x}}_{\ell_{3}=\ell_{1},j}^{\text{mRNA}} & = & \sum_{i_{1}}{\tilde{x}}_{\ell_{1},i_{1}}^{\text{mRNA}}x_{i_{1},j},\\ {\tilde{x}}_{\ell_{3}=\ell_{2},j}^{\text{miRNA}} & = & \sum_{i_{2}}{\tilde{x}}_{\ell_{2},i_{2}}^{\text{miRNA}}x_{i_{2},j}, \end{array}$$

The first five are shown separately in (Fig 1(b) and 1(c)). It is obvious that all of the ten sample singular value vectors are significantly coincident with sample classifications.

**Fig 7:**
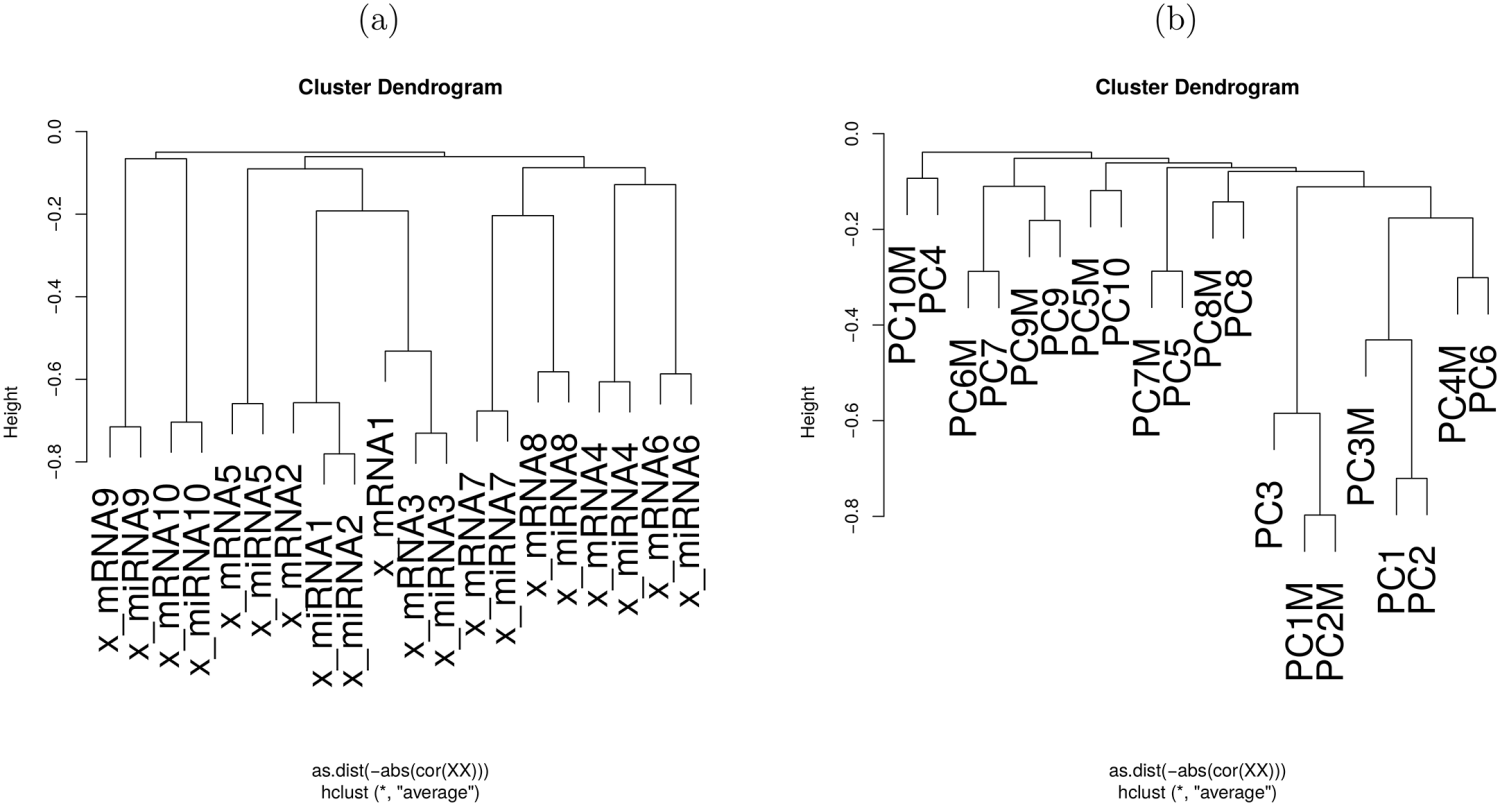
Hierarchical clustering of ${\tilde{x}}_{\ell_{3},j}^{\text{mRNA}}$(x_mRNA) and ${\tilde{x}}_{\ell_{3},j}^{\text{miRNA}}$ (x_miRNA). When TD was applied to type II tensor (a) and *v*_*ℓ*_3_,*j*_ (for mRNA, labelled as PC), and $v_{\ell_{3},j}^{\prime }$ (for miRNA, labelled as PCM) when PCA was separately applied to miRNA and mRNA (b) (1 ≤ *ℓ*_3_ ≤ 10). Distances were negative signed absolute values of Pearson correlation coefficients. Unweighted Pair Group Method with Arithmetic mean (UPGMA) was employed.

To determine whether TD applied to type II tensors could depict latent correlation between miRNA and mRNA, hierarchical clustering was performed between ${\tilde{x}}_{\ell_{3},j}^{\text{mRNA}}$ and ${\tilde{x}}_{\ell_{3},j}^{\text{miRNA}}$ (1 ≤ *ℓ*_3_ ≤ 10, Fig 7(a)). Here, ${\tilde{x}}_{\ell_{3},j}^{\text{mRNA}},3\leq \ell_{3}\leq 10$ are always paired with one of ${\tilde{x}}_{\ell_{3},j}^{\text{miRNA}},3\leq \ell_{3}\leq 10$ (Fig 7(a)). Thus, TD applied to type II tensor could successfully identify latent correlation between two views, i.e., mRNA and miRNA.

Additionally, I attempted to extract mRNAs and miRNAs using the first five mRNA and miRNA singular value vectors, ${\tilde{x}}_{\ell_{1},i_{1}}^{\text{mRNA}}$,${\tilde{x}}_{\ell_{2},i_{2}}^{\text{miRNA}}$, and 1 ≤ *ℓ*_1_, *ℓ*_2_ ≤ 5, respectively. In this example two views were emplyed, as the type II tensor is occasionally the matrix ${\tilde{x}}_{i_{1},i_{2}}$. Thus, the core tensor, $\tilde{G}\left(\ell_{1},\ell_{2}\right)$, is the diagonal matrix. Therefore, the first five feature singular value vectors are automatically associated with the first five corresponding sample singular value vectors. 21 miRNAs (let-7a/b/f, miR-103/125b/141/142-3p/143/145/148a/199a/b-3p/19b/205/21/22/23a/24/26a/30a/451/99a), identified as outliers using the first five miRNA singular value vectors, ${\tilde{x}}_{\ell_{2},i_{2}},1\leq \ell_{2}\leq 5$, were selected. Eight miRNAs (let-7a, miR-21/22/451/142-3p/143/145/99a) were also reported in the original study ([30], Table 1). Three hundred seventy-four mRNA probes, identified as outliers using the first five mRNA singular value vectors ${\tilde{x}}_{\ell_{1},i_{1}}^{\text{mRNA}},1\leq \ell_{1}\leq 5$, were selected (associated mRNAs are in S1 Table) and were substantially overlapped with those selected when type I tensors were considered (Table 4). In order to evaluate obtained mRNAs and miRNAs biologically, mRNAs associated with 374 probes were uploaded to MSigDB, and miRNAs to DIANA-mirpath. DIANA-mirpath identified “MiRNAs in cancer” as the most significant KEGG pathway (Table 3). Table 2 also shows the overlap with MSigDB. Eight out of ten were breast cancer related, and one of the remaining two is related to metastasis. Thus, these identified mRNAs and miRNAs are biologically reasonable. In conclusion, TD applied to type II tensor works well.

**Table 4:** Comparison between 426 mRNA probes identified by TD based unsupervised FE applied to type I tensor and 374 mRNA probes identified by TD based unsupervised FE applied to type II tensor, or 427 probes identified by PCA based unsupervised FE separately applied to miRNA/mRNA. S:selected, NS:not selected.

		TD type II		PCA	
		NS	S	NS	S
TD	NS	12856	110	12948	19
type I	S	163	264	18	408

### Temporally differentially expressed genes

Although the application of TD based unsupervised FE to multi-omics data was successful, one may wonder if the application to muti-omics data is reasonable, as synthetic datasets were more difficult to deal with due to a lack of class labelling. Have I intentionally tried the easiest case? In order to address this possible query, I considered two examples of a more difficult problem: identification of temporally differentially expressed genes. The task is as follows: Given more than one temporal gene expression, identify genes expressed differently among multiple expressions at specific time points. For example, expression of a particular gene obeys *f*(*t*) as a function of *t* under one set of conditions, while it obeys *f*(*t*) + *C* with a constant *C* under another. Conventionally, this is differentially expressed between the two conditions; however this kind of differential expression is often not of interest when temporal gene expression is considered. One distinction of note would be, for example, differences between expressions at certain time points and not at others (a distinct time dependency between two conditions). However, there are no *de facto* standard methods that automatically achieve this. In order to address it, TD based unsupervised FE was applied to this problem.

The first example of this application is the comparison of non-small lung cancer cell (NSCLC) line H1975, with and without EGF treatment [33]. Gene expression matrices were divided into two groups (with and without EGF treatment). The type I tensor *x*_*t*_1_,*t*_2_,*i*_ is then generated as

$$\begin{array}{c} x_{t_{1},t_{2},i}=x_{i,t_{1}}^{\text{control}}x_{i,t_{2}}^{\text{EGF}} \end{array}$$

where $x_{i,t_{1}}^{\text{control}}$ and $x_{i,t_{2}}^{\text{EGF}}$ are *i*th gene expressions of cell lines with and without EGF treatment, at time points *t*_1_ and *t*_2_ after the EGF or control treatments. As they share features (though not samples) in contrast to the previous application which was Case I data, this example uses Case II data. As the samples in this example are divided into two groups based on EGF treatment, it is not fully unsupervised. It is, however, unsupervised in the sense that the type of temporal difference sought is not defined. The tensor was expanded by HOSVD as

$$\begin{array}{c} x_{t_{1},t_{2},i}=\sum_{\ell_{1}}\sum_{\ell_{2}}\sum_{\ell_{3}}G\left(\ell_{1},\ell_{2},\ell_{3}\right)x_{\ell_{1},t_{1}}^{\text{control}}x_{\ell_{2},t_{2}}^{\text{EGF}}x_{\ell_{3},i} \end{array}$$

Fig 2(a) and 2(b) shows the $x_{\ell_{1},t_{1}}^{\text{control}}$ and $x_{\ell_{2},t_{2}}^{\text{EGF}}$ for *ℓ*_1_, *ℓ*_2_ = 1, 2, respectively. Obviously, those for *ℓ*_1_ = *ℓ*_2_ = 1 do not have any time dependence while those for *ℓ*_1_ = *ℓ*_2_ = 2 do. Some temporal difference was observed in the latter, however its significance is unclear. In order to determine said significance, genes identified as outliers had to be selected. This selection process began with identifying the gene singular value vectors associated with $x_{\ell_{1}=2,t_{1}}^{\text{control}}$ and $x_{\ell_{2}=2,t_{2}}^{\text{EGF}}$. Table 1 shows the top ranked *G*(*ℓ*_1_, *ℓ*_2_, *ℓ*_3_)s with larger absolute values. It is obvious that *x*_*ℓ*_3_ = 2,*i*_ is associated with $x_{\ell_{1}=2,t_{1}}^{\text{control}}$ and $x_{\ell_{2}=2,t_{2}}^{\text{EGF}}$, as the absolute values of *G*(2, 2, 1) and *G*(2, 1, 2) are the second and the third largest in the table. Next, 558 mRNA probes (associated mRNAs are in S1 Table) identified as outliers based on *x*_*ℓ*_3_ = 2,*i*_ were selected. Fig 2(c) shows the histogram of correlation coefficients between the vectors generated by connecting $x_{\ell_{1},t_{1}}^{\text{control}}$ and $x_{\ell_{2},t_{2}}^{\text{EGF}}$ vs the 558 selected mRNA probes. These are highly correlated (adjusted *P*-values are less than 0.01). It is remarkable since *G*(2, 2, 1) and *G*(2, 1, 2) are smaller than one tenth of *G*(1, 1, 1) whose absolute value is the largest. This suggests that the amount of contributions of *G*(2, 2, 1) and *G*(2, 1, 2) is too little to govern individual gene expression. The high correlation despite this fact speaks to the soundness of our methodology.

The next step was to determine whether the 558 mRNA probes selected exhibit temporal differences. The 558 mRNA probes selected are scaled, shifted, and over drawn as boxplot (Fig 2(d)). Though it is difficult to observe, *P*-values were computed by two-sided *t* tests between expressions, with and without EGF treatments at time points, 0.5, 1, 2, 4, 6 and 48 hours (Fig 2). These values are significant only at limited time points with and without EFG treatment. These were merely temporally differently expressed genes. Thus, TD based unsupervised FE applied to type I tensors is effective.

To determine the biological reliability of the selected genes, genes associated with selected 558 mRNA probes were uploaded to MSigDB. The second significant gene set was found to be KOBAYASHI_EGFR_SIGNALING_24HR_DN (the adjusted *P* value is 1.37 × 10^−96^), which is reasonable as the genes sought were expressed differently with and without EGF treatments.

The next task was to determine whether type II tensors produce similar results. Type II tensor ${\tilde{x}}_{t_{1},t_{2}}$, which in this example is the matrix since two views are considered, is defined as a summation of a type I tensor over gene index,

$$\begin{array}{ccc} {\tilde{x}}_{t_{1},t_{2}} & = & \sum_{i}x_{t_{1},t_{2},i} \end{array}$$

that is expanded by HOSVD, which is a simple SVD in the present case

$$\begin{array}{ccc} {\tilde{x}}_{t_{1},t_{2}} & = & \sum_{\ell_{1}}\sum_{\ell_{2}}\tilde{G}\left(\ell_{1},\ell_{2}\right){\tilde{x}}_{\ell_{1},t_{1}}^{\text{control}}{\tilde{x}}_{\ell_{2},t_{2}}^{\text{EGF}} \end{array}$$

Fig 2(e) and 2(f) shows the ${\tilde{x}}_{\ell_{1},t_{1}}^{\text{control}}$ and ${\tilde{x}}_{\ell_{2},t_{2}}^{\text{EGF}}$ for *ℓ*_1_, *ℓ*_2_ = 1, 2. Obviously, ${\tilde{x}}_{\ell_{1}=1,t_{1}}^{\text{control}}$ and ${\tilde{x}}_{\ell_{2}=1,t_{2}}^{\text{EGF}}$ do not have any time dependence while ${\tilde{x}}_{\ell_{1}=2,t_{1}}^{\text{control}}$ and ${\tilde{x}}_{\ell_{2}=2,t_{2}}^{\text{EGF}}$ do, as in the case where TD was applied to a type I tensor. Some temporal difference was observed between ${\tilde{x}}_{\ell_{1}=2,t_{1}}^{\text{control}}$ and ${\tilde{x}}_{\ell_{2}=2,t_{2}}^{\text{EGF}}$. Again, the significance of this temporal difference was unclear. Genes identified as outliers had to be selected to determine the significance of this temporal difference. While there are two gene singular value vectors,

$$\begin{array}{c} {\tilde{x}}_{\ell_{3}=\ell_{1},i}^{\text{control}}=\sum_{t_{1}}{\tilde{x}}_{\ell_{1},t_{1}}^{\text{control}}x_{i,t_{1}},\;\;{\tilde{x}}_{\ell_{3}=\ell_{2},i}^{\text{EGF}}=\sum_{t_{2}}{\tilde{x}}_{\ell_{2},t_{2}}^{\text{EGF}}x_{i,t_{2}}, \end{array}$$

485 and 471 mRNA probes identified as outliers were selected using ${\tilde{x}}_{\ell_{3}=\ell_{1},i}^{\text{control}}$ and ${\tilde{x}}_{\ell_{3}=\ell_{2},i}^{\text{EGF}}$, respectively, among which 398 mRNA probes (associated mRNAs are shown in S1 Table) were commonly selected. Fig 2(g) shows the histogram of correlation coefficients between the vector generated by connecting ${\tilde{x}}_{\ell_{1},t_{1}}^{\text{control}}$ and ${\tilde{x}}_{\ell_{2},t_{2}}^{\text{EGF}}$ vs 398 commonly selected mRNA probes. These were highly correlated (adjusted *P*-values are less than 0.01). This is noteworhty as the smallest contribution from the second singular value vector was 1 × 10^−5^. This suggests that the amount of contributions of the second singular value vector were too small to govern individual gene expressions. As before, the high correlation despite this fact speaks to the soundness of our methodology.

The next task was to determine whether the genes selected exhibit temporal difference. The genes selected are scaled, shifted, and over drawn as boxplot (Fig 2(h)). Though it is difficult to observe, *P*-values computed by a two-sided *t* test between expression at time points, 0.5, 1, 2, 4, 6 and 48 hours (Fig 2) are significant only at limited time points with and without EFG treatment. Although this is of less significance than TD applied to a type I tensor, these are merely temporally differently expressed genes. Thus, TD based unsupervised FE applied to type II tensor is effective.

In order to see the biological reliability of selected genes, the mRNAs associated with commonly selected 398 mRNA probes were uploaded to MSigDB. The second most significant gene set was determined to be KOBAYASHI_EGFR_SIGNALING_24HR_DN (the adjusted *P* value is 1.37 × 10^−128^), which, again, was reasonable as the genes sought expressed differently with and without EGF treatments. Although the number of genes selected was less than that by TD applied to type a I tensor, since the *P*-value is smaller, the significance was greater than that of TD applied to a type I tensor.

As a whole, both TD applied to type I type II tensors is effective.

The next temporally differentially expressed gene detection example is vaccine infection experiment [34]. Patients were divided into three groups, P, D and NP. As sample classification was used, it is also not fully unsupervised. It is, however, unsupervised in the sense that no temporal functional forms are assumed. $x_{i,t_{1}}^{\text{P}}$, $x_{i,t_{2}}^{\text{D}}$, and $x_{i,t_{3}}^{\text{NP}}$ are the *i*th gene expressions of protected, delayed, and non-protected, patients at time points *t*_1_, *t*_2_ and *t*_3_ after vaccine treatments, respectively. Type I tensor *x*_*t*_1_,*t*_2_,*t*_3_,*i*_, was defined as

$$\begin{array}{c} x_{t_{1},t_{2},t_{3},i}=x_{i,t_{1}}^{\text{P}}x_{i,t_{2}}^{\text{D}}x_{i,t_{3}}^{\text{NP}} \end{array}$$

*x*_*t*_1_,*t*_2_,*t*_3_,*i*_ would be expanded by HOSVD as

$$\begin{array}{c} x_{t_{1},t_{2},t_{3},i}=\sum_{\ell_{1},\ell_{2},\ell_{3},\ell_{4}}G\left(\ell_{1},\ell_{2},\ell_{3},\ell_{4}\right)x_{\ell_{1},t_{1}}^{\text{P}}x_{\ell_{2},t_{2}}^{\text{D}}x_{\ell_{3},t_{3}}^{\text{NP}}x_{\ell_{4},i}, \end{array}$$

however, the total memory required to store all of this expansion is too large to be prepared. Fortunately, as can be seen in the application to the first temporally differentially expressed gene identification, TD applied to a type II tensor that requires much smaller (1/*N*) memory can be as effective as TD applied to type I tensor for temporally differentially expressed gene identification. Thus, for this example, I employ only type II tensor ${\tilde{x}}_{t_{1},t_{2},t_{3}}$, defined as

$$\begin{array}{c} {\tilde{x}}_{t_{1},t_{2},t_{3}}=\sum_{i}x_{t_{1},t_{2},t_{3},i} \end{array}$$

that can be expanded as

$$\begin{array}{c} {\tilde{x}}_{t_{1},t_{2},t_{3}}=\sum_{\ell_{1}}\sum_{\ell_{2}}\sum_{\ell_{3}}\tilde{G}\left(\ell_{1},\ell_{2},\ell_{3}\right){\tilde{x}}_{\ell_{1},t_{1}}^{\text{P}}{\tilde{x}}_{\ell_{2},t_{2}}^{\text{D}}{\tilde{x}}_{\ell_{3},t_{3}}^{\text{NP}} \end{array}$$

by HOSVD.

Fig 3(a) and 3(b) shows the ${\tilde{x}}_{\ell_{1},t_{1}}^{\text{P}}$, ${\tilde{x}}_{\ell_{2},t_{2}}^{\text{D}}$ and ${\tilde{x}}_{\ell_{3},t_{3}}^{\text{NP}}$ for *ℓ*_1_,*ℓ*_2_ = 1, 2. Obviously, ${\tilde{x}}_{\ell_{1}=1,t_{1}}^{\text{P}}$, ${\tilde{x}}_{\ell_{2}=1,t_{2}}^{\text{D}}$ and ${\tilde{x}}_{\ell_{3}=1,t_{3}}^{\text{NP}}$ do not have any time dependence while those for *ℓ*_1_ = *ℓ*_2_ = *ℓ*_3_ = 2 do, as in the EGF treated cell line cases. Though ${\tilde{x}}_{\ell_{1}=2,t_{1}}^{\text{P}}$, ${\tilde{x}}_{\ell_{2}=2,t_{2}}^{\text{D}}$ and ${\tilde{x}}_{\ell_{3}=2,t_{3}}^{\text{NP}}$ also seem to have some temporal difference, its significance is again unclear. To determine the significance of this difference, genes identified as outliers had to be selected. There are three gene singular value vectors:

$$\begin{array}{ccc} {\tilde{x}}_{\ell_{4}=\ell_{1},i}^{\text{P}} & = & \sum_{t_{1}}{\tilde{x}}_{\ell_{1},t_{1}}^{\text{P}}x_{i,t_{1}}^{\text{P}},\\ {\tilde{x}}_{\ell_{4}=\ell_{2},i}^{\text{D}} & = & \sum_{t_{2}}{\tilde{x}}_{\ell_{2},t_{2}}^{\text{D}}x_{i,t_{2}}^{\text{D}}\\ {\tilde{x}}_{\ell_{4}=\ell_{3},i}^{\text{NP}} & = & \sum_{t_{3}}{\tilde{x}}_{\ell_{3},t_{3}}^{\text{NP}}x_{i,t_{3}}^{\text{NP}} \end{array}$$

Using these three gene singular value vectors with *ℓ*_1_ = *ℓ*_2_ = *ℓ*_3_ = 2, 104 mRNA probes identified commonly as outliers were selected (S1 Table).

Fig 3(c) shows the histogram of correlation coefficients between the vector generated by connecting ${\tilde{x}}_{\ell_{1},t_{1}}^{\text{P}}$, ${\tilde{x}}_{\ell_{2},t_{2}}^{\text{D}}$ and ${\tilde{x}}_{\ell_{3},t_{3}}^{\text{D}}$ vs selected 104 mRNA probes. These are highly correlated (adjusted *P*-values are less than 0.01). This is noteworthy as $\tilde{G}\left(1,2,2\right),\tilde{G}\left(2,1,2\right),\tilde{G}\left(2,2,1\right)$, being the three largest core tensors associated with these three gene singular value vectors, had the contributions as small as 1 × 10^−3^ of $\tilde{G}\left(1,1,1\right)$, the largest one. This suggests that the amount of contributions of the second gene singular value vector is too small to govern individual gene expression. The high correlation despite this fact suggests the soundness of our methodology.

The next task was to determine whether the mRNA probes selected exhibit temporal difference. The mRNA probes selected are scaled, shifted, and overdrawn as boxplot (Fig 3(d)). Though it is difficult to observe, *P*-values computed by categorical regression assuming three classes (P, D, NP) at time points, 1, 2, 3, 4 and 5 days (Fig 3) are significant at all time points between the three classes. This was clearly not due to simple baseline shifts, as can be seen in Fig 3(d). This is due to as temporally differently expressed genes. Thus, TD based unsupervised FE applied to type II tensor is also effective.

In order to determine the biological reliability of selected genes, the mRNAs associated with the selected 104 mRNA probes were uploaded to MSigDB. The top six significant gene sets were determined to be: GSE13485_X_VS_Y_YF17D_VACCINE_PBMC_DN, GSE10325_MYELOID_VS_LUPUS_MYELOID_DN, GSE13485_X_VS_Y_YF17D_VACCINE_PBMC_DN, GSE13485_X_VS_Y_YF17D_VACCINE_PBMC_DN, GSE13485_X_VS_Y_YF17D_VACCINE_PBMC_DN, and GSE13485_X_VS_Y_YF17D_VACCINATION_PBMC_DN, where (X, Y) = (CTRL, DAY7), (DAY1, DAY7), (CTRL, DAY3), (DAY3, YF17D), and (PRE, POST) in this order, which are associated with adjusted *P*-values, 2.97 × 10^−66^, 2.98 × 10^−57^, 3.69 × 10^−55^, 4.86 × 10^−53^, 5.43 × 10^−51^, and 6.36 × 10^−49^, respectively. As five out of six are related to vaccination, TD based unsupervised FE selected biologically feasible sets of genes.

In conclusion, TD based unsupervised FE is also effective also for the identification of temporally differentially expressed genes.

## Discussion

In the following section I discuss the strategy for applying TD based unsupervised FE to tensors built from matrix products, methodological points of view, and outcomes obtained by applying this strategy to multi-omics datasets from the biological point of views.

### Comparisons with various methods applicable to synthetic data

The synthetic datasets that presented in the above sections are very difficult to analyse using standard supervised statistical analysis methods. In the supervised methodology, all background knowledge of given datasets is required, e.g., classification labels or assumed functional forms (for example, monotonic increase/decrease or periodicity). Alternatively, TD applied to type I tensors is Eq (3) and applied to type II tensor is Eq (4), which can be performed in the synthetic dataset prepared without any information in advance. To my knowledge, there are no applicable supervised methodologies for the synthetic datasets presented above. Thus, in the following I discuss only unsupervised methods.

As the first *N*_0_ features are derived from the common bases shown in Fig 4(a)–4(c), it is possible to detect them by computing correlations between them. However, as can be seen in Fig 4(d)–4(f), the correlations are highly non-linear, and it is therefore impossible to detect them (in actuality, the Pearson correlation between two variables shown is Fig 4(f) is as small as -0.01).

One may wonder if correlation analysis considering linear combinations, e.g., canonical correlation analysis (CCA), can depict latent correlation. However, in CCA, as *M* dimensional vectors as numerous as *N* must be compared, it is an overcomplete problem when *M* < *N* (and this is the present case). Thus, canonical correlation coefficients generated from linear combinations are always 1.0. This means that there is no way to detect latent correlation between *N*_0_(<*N*) features.

**Fig 8:**
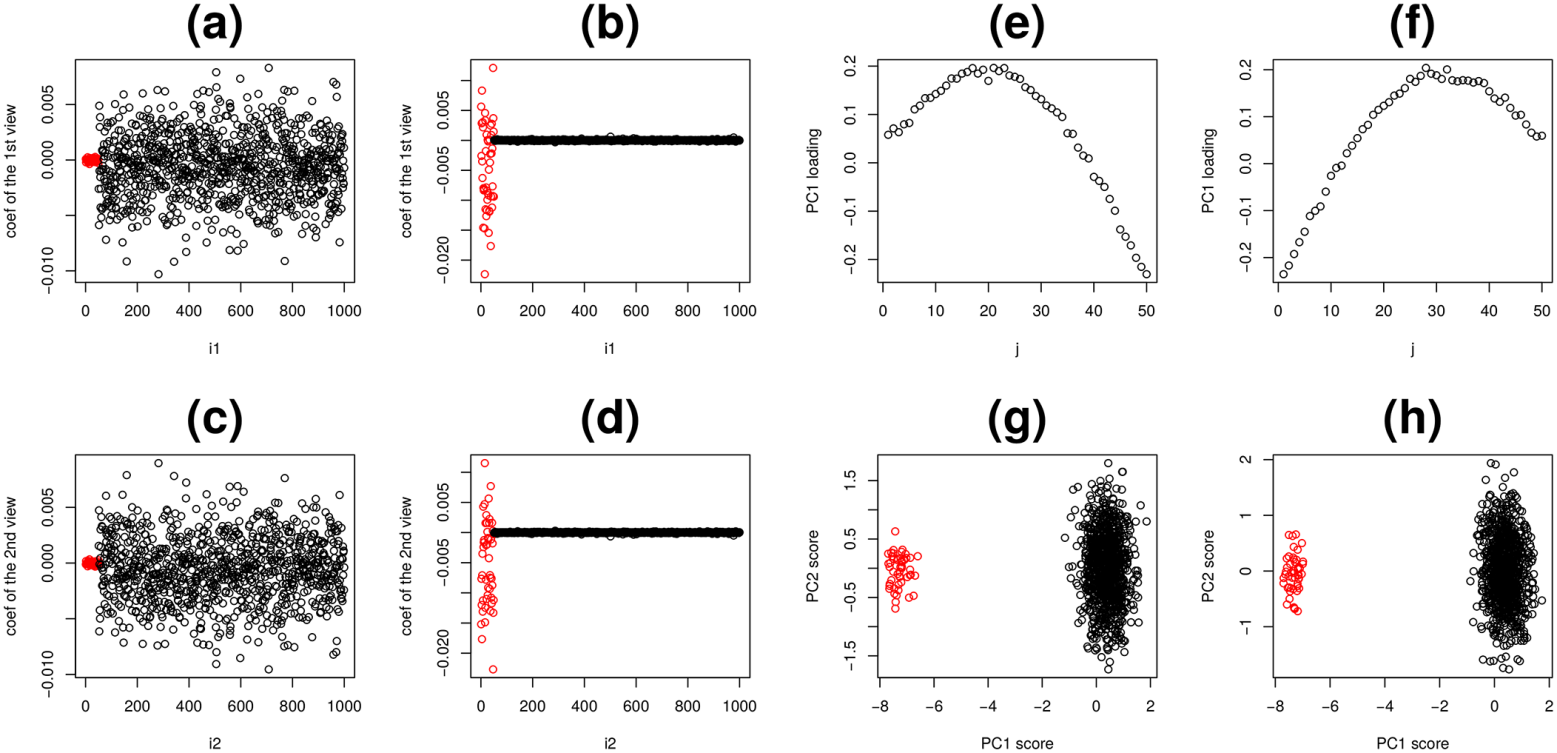
Two alternative methods applied to synthetic data. (a) to (d): The results of kCCA. Vertical axes are the coefficients used for linear combinations of *N* features, Horizontal axes are *i*_1_ and *i*_2_, i.e., indices attributed to *N* features. (a) and (b): the first view, (c) and (d) the second view. (a) and (c): the first type of kCCA results and (b) and (d): the second type of kCCA results. The distinction between the two types of kCCA results is coincident with the distinction between features with latent correlation (1 ≤ *j* ≤ *N*_0_) and those without correlation (*N*_0_ < *j* ≤ *N*). However, as computed correlation coefficients were as high as 0.99, kCCA failed to identify latent correlation. (e) to (h): PCA separately applied to two views in synthetic data. (e) and (f): the first PC loadings attributed to *M* samples in each view. (g) and (h) the first and the second PC scores attributed to *N* features in each view. Red open circles are features with latent correlation (1 ≤ *j* ≤ *N*_0_). Black circles are those composed of random numbers (*N*_0_ < *j* ≤ *N*).

Similar problems stand for nonlinear correlation analysis like kernel CCA (kCCA). KCCA was applied to matrices *X*^(*k*)^, *k* = 1, 2 in the above synthetic examples. The ten components (this is the kCCA default) generated are classified into two types, each of which are distinct from the first *N*_0_ features and remaining (Fig 8(a)–8(d)). Thus, the correlation is apparently successful, however, both results in the correlation coefficients were as large as 1.0 meaning that kCCA evaluated the correlation of two views between the first *N*_0_ features and that between remaining features the latter composed of simple random numbers as demonstrated in the above. Thus, kCCA cannot distinguish between latent correlation between the first *N*_0_ features and random numbers, and cannot be successfully applied.

Finally, PCA based unsupervised FE, which was recently proposed and successfully applied to various integrated analyses of multi-omics datasets, was again applied here. As PCA is equivalent to singular value decomposition (SVD),

$$\begin{array}{c} x_{i_{1},j}^{\left(1\right)}=\sum_{\ell_{1}}u_{\ell_{1},i_{1}}\lambda_{\ell_{1}}^{1/2}v_{\ell_{1},j},\;\;x_{i_{2},j}^{\left(2\right)}=\sum_{\ell_{2}}u_{\ell_{2},i_{2}}^{\prime }{\lambda^{\prime }}_{\ell_{2}}^{1/2}v_{\ell_{2},j}^{\prime } \end{array}$$

Fig 8(e) and 8(f) are *v*_*ℓ*_1_ = 1, *j*_ and $v_{\ell_{2}=1,j}^{\prime }$, respectively. *u*_*ℓ*_1_,*i*_1__,*ℓ*_1_ = 1, 2 is Fig 8(g) and *u*_*ℓ*_2_,*i*_2__, *ℓ*_2_ = 1, 2 is Fig 8(h). *λ*_*ℓ*_2__ and *λ*_*ℓ*_3__ are the eigen values computed with PCA. Thus

$$\begin{array}{c} x_{i_{1},j}^{\left(1\right)}x_{i_{2},j}^{\left(2\right)}=\sum_{\ell_{1}}\sum_{\ell_{2}}v_{\ell_{1},j}\lambda_{\ell_{1}}^{1/2}{\lambda^{\prime }}_{\ell_{2}}^{1/2}v_{\ell_{2},j}^{\prime }u_{\ell_{1},i_{1}}u_{\ell_{2},i_{2}}^{\prime } \end{array}$$

Compared with Eq (3), if

$$\begin{array}{ccc} \sum_{\ell_{3}}G\left(\ell_{1},\ell_{2},\ell_{3}\right)x_{\ell_{3},j} & = & v_{\ell_{1},j}\lambda_{\ell_{1}}^{1/2}{\lambda^{\prime }}_{\ell_{2}}^{1/2}v_{\ell_{2},j}^{\prime }\\ x_{\ell_{1},i_{1}} & = & u_{\ell_{1},i_{1}},\\ x_{\ell_{2},i_{2}} & = & u_{\ell_{2},i_{2}}^{\prime } \end{array}$$

and PCA is equivalent to TD applied to a a type I tensor. Alternatively, compared with Eq (4), if

$$\begin{array}{ccc} \tilde{G}\left(\ell_{1},\ell_{2}\right) & = & \lambda_{\ell_{1}}^{1/2}{\lambda^{\prime }}_{\ell_{2}}^{1/2}\sum_{j}v_{\ell_{1},j}v_{\ell_{2},j}^{\prime } \end{array}$$ (5)

$$\begin{array}{ccc} {\tilde{x}}_{\ell_{1},i_{1}}^{\left(1\right)} & = & u_{\ell_{1},i_{1}}\\ {\tilde{x}}_{\ell_{2},i_{2}}^{\left(2\right)} & = & u_{\ell_{2},i_{2}}^{\prime } \end{array}$$

then PCA is equivalent to TD applied to a type II tensor. However, HOSVD does not always produce the solution satisfying the above. For example, since Eq (4) computed with HOSVD a standard SVD, $\tilde{G}\left(\ell_{1},\ell_{2}\right)$ is diagonal, while Eq (5) cannot be diagonal since *v*_*ℓ*_1_,*j*_ is not orthogonal to $v_{\ell_{2},j}^{\prime }$.

Although PCA based unsupervised FE successfully identified the first *N*_0_ features associated with latent correlations as outliers (Fig 8(g) and 8(h)), PC loadings attributed to samples (Fig 8(e) and 8(f)) are almost identical to Fig 4(d) and 4(e), which are not correlated (Fig 4(f)). Therefore PCA based unsupervised FE cannot identify latent correlation. In the previous applications of PCA based unsupervised FE aimed at integrated analysis of multi-omics data [14, 22], it was critical to identify pairs of highly correlated PC loadings, otherwise it was not possible to identify which PCs should be used for FE. In this context, PCA based unsupervised FE failed to detect latent correlations among multi-views.

While PCA was unsuccessful, HOSVD was applied to type I tensors in an attempt to decompose *v*_*ℓ*_1_,*j*_ and $v_{\ell_{2},j}^{\prime }$ (Fig 8(e) and 8(f)), which are not orthogonal, into orthogonal bases *x*_*ℓ*_3_,*j*_ as shown in Fig 4(g)–4(i). For this reason, TD applied to type I tensors is superior to PCA when applied to the matrix products of synthetic datasets and can depict latent correlation that PCA failed to identify. As for HOSVD applied to type II tensors, Fig 6(b) and 6(e) correspond to Fig 4(e) while Fig 6(c) and 6(f) correspond to Fig 4(d) shows HOSVD applied to type II tensors can depict the latent correlation that PCA based unsupervised FE failed to detect.

### Methodological discussion of TD based unsupervised FE applied to multi-omics data

In contrast to synthetic data (to which no supervised methods apply), the supervised method can be applied to the multi-omics data used in this study can be treated with supervised method as the data uses class labels. Strictly speaking, there are no unsupervised methods applicable to multi-view data processing other than TD based unsupervised FE. We can, therefore, even conclude that is the above strategy is sound. That said, it would be beneficial to demonstrate that the unsupervised methodology is superior to the supervised methodology.

As mentioned in above, most multi-view data processing methodologies require optimization of weights. I do not consider such methodologies, since optimizing weights is a complicated unnecessary process. Therefore, they are not comparable with the unsupervised strategy, which involves no parameter optimization processes.

Thus, the multi-view data processing methodology is considered applicable to merged matrices shown in Eqs (1) or (2), which does not include any weight optimization. Here, I consider three alternative methodologies, Significance Analysis of Microarrays (SAM), [35] Limma [36] and randomforest [37] (RF). As SAM and Limma evaluate features independently, weights attributed to views are not required. Although RF evaluates features in more collective ways, the evaluations are tree based, therefore the absolute values of each feature are not important. Table 5 shows the pertinent results. All three methods are inferior to the methodology presented above, as they failed to identify a significantly small number of features. Limma selected all of 755 miRNAs as significant. Roughly speaking, at least half of mRNAs were identified by these three methods.

**Table 5:** The numbers of identified mRNAs and miRNAs (multi-omics) and mRNAs (vaccination) using various methodologies. Multi-omics: Among 427 mRNA probes and 12 miRNAs identified PCA based unsupervised FE, 408 mRNA probes (Table 4) and 9 miRNAs were also identified with TD based unsupervised FE applied to type I tensor.

	SAM	Limma	RF	PCA based unsupervised FE
multi-omics				
mRNA	8055	6055	5079	427
miRNA	148	755	186	12
vaccine				
mRNA	11739	9445	8300	18

Although these might be enough to demonstrate the superiority of the methodology presented above, measures were also undertaken to reduce the number of mRNAs identified by reducing threshold *P*-values ensuring that these three methods identify 426 mRNA probes, which is the same number identified by TD applied to type I tensors. As threshold *P*-values which are too small without any statistical justifications may not be acceptable, in order to evaluate these three methods, unnatural threshold *P*-values were intentionally used. Top ranked mRNAs selected by using intentionally reduced threshold *P*-values were uploaded to MSigDB server. However, breast cancer was rarely identified (Table 6). Breast cancer was identified only once by SAM, and was not detected in either limma or RF. These outcomes are in contrast to Table 2, where eight out of ten significant gene sets are breast cancer-related when TD was applied to either type I or type II tensor. Thus, it is obvious that these methodologies are inferior to the methodology presented above in the present study, i.e., TD based unsupervised FE.

**Table 6:** Top 10 significant overlap gene set in MSigDB with top ranked 400 (approx) mRNA probes identified by alternative methods SAM, limma, and RF, as well as 374 mRNA probes identified by HO GSVD. “BREAST_CANCER” was presented in bold to emphasize the overlap with breast cancer, whose counts are in parentheses at the right side of method names.

	SAM (1)	limma (0)	RF (0)	HO GSVD (5)
1	BLALOCK_ALZHEIMERS_DISEASE_UP	PUJANA_BRCA1_PCC_NETWORK	DIAZ_CHRONIC_MEYLOGENOUS_LEUKEMIA_UP	SMID_**BREAST_CANCER**_LUMINAL_B_DN
2	SMID_**BREAST_CANCER**_NORMAL_LIKE_UP	BLALOCK_ALZHEIMERS_DISEASE_UP	BLALOCK_ALZHEIMERS_DISEASE_UP	SMID_**BREAST_CANCER**_BASAL_DN
3	SWEET_LUNG_CANCER_KRAS_DN	KINSEY_TARGETS_OF_EWSR1_FLII_FUSION_UP	HAMAI_APOPTOSIS_VIA_TRAIL_UP	YOSHIMURA_MAPK8_TARGETS_UP
4	WEST_ADRENOCORTICAL_TUMOR_DN	RODRIGUES_THYROID_CARCINOMA_POORLY_DIFFERENTIATED_UP	PUJANA_BRCA1_PCC_NETWORK	SMID_**BREAST_CANCER**_RELAPSE_IN_BONE_DN
5	BOQUEST_STEM_CELL_CULTURED_VS_FRESH_UP	PUJANA_CHEK2_PCC_NETWORK	GRAESSMANN_APOPTOSIS_BY_DOXORUBICIN_DN	JAEGER_METASTASIS_DN
6	PUJANA_BRCA1_PCC_NETWORK	DIAZ_CHRONIC_MEYLOGENOUS_LEUKEMIA_UP	MILI_PSEUDOPODIA_HAPTOTAXIS_UP	GOZGIT_ESR1_TARGETS_DN
7	GRAESSMANN_APOPTOSIS_BY_DOXORUBICIN_DN	GRAESSMANN_APOPTOSIS_BY_DOXORUBICIN_DN	BUYTAERT_PHOTODYNAMIC_THERAPY_STRESS_UP	SMID_**BREAST_CANCER**_BASAL_UP
8	HAMAI_APOPTOSIS_VIA_TRAIL_UP	RODRIGUES_THYROID_CARCINOMA_ANAPLASTIC_UP	PUJANA_ATM_PCC_NETWORK	ONDER_CDH1_TARGETS_2_DN
9	BUYTAERT_PHOTODYNAMIC_THERAPY_STRESS_UP	FEVR_CTNNB_TARGETS_DN	PUJANA_CHEK2_PCC_NETWORK	SENGUPTA_NASOPHARYNGEAL_CARCINOMA_WITH_LMP1_DN
10	PUJANA_CHEK2_PCC_NETWORK	CAIRO_HEPATOBLASTOMA_CLASSES_UP	BLALOCK_ALZHEIMERS_DISEASE_DN	FARMER_**BREAST_CANCER**_APOCRINE_VS_LUMINAL

Finally, PCA based unsupervised FE was applied to mRNAs and miRNAs separately (Table 5). PCA based unsupervised FE identified smaller numbers of mRNAs and miRNAs than the above three methodologies. Especially, since mRNAs selected are almost identical to those identified by TD based unsupervised FE applied to type I tensor (Table 4), PCA based unsupervised FE is as effective for the identification of biologically reliable mRNAs. However, it cannot identify latent correlation between miRNAs and mRNAs, as hierarchical clustering of PC loading attributed to samples identified no pairs of miRNAss and mRNA (Fig 7(b)), which were identified when TD applied to type II tensors was considered and without which no integrated analysis of multi-omics datasets by PCA based unsupervised FE were successful. This indicates that the present datasets were more complex and cannot be dealt with using PCA based unsupervised FE in an integrated manner. Thus, I conclude that TD based unsupervised FE applied to type I or type II tensors is the only method for achieving two tasks: (i) identifying sufficiently small numbers of biologically important features, and (ii) identify latent correspondence between multi-omics profiles.

Here, PCA based unsupervised FE is shown to be the only strategy that can compute with the TD based unsupervised FE applied to matrix products. A more detailed comparison of these two strategies may enable us to understand the functionality of TD based unsupervised FE. As can be seen in the application to synthetic data, TD applied to type I tensors attempted to decompose *v*_*ℓ*_1_,*j*_ and *v*′_*ℓ*_2_,*j*_ into orthogonal bases *x*_*ℓ*_3_,*j*_. This also occurred in the application to multi-omics datasets. First, I identified that the first miRNA PC loading, $v_{\ell_{2}=1,j}^{\prime }$, dominates subsequent PC loadings (more than 80%). Next, I also identified that the first mRNA PC loadings attributed to samples *v*_*ℓ*_1_,*j*_ and 1 ≤ *ℓ*_1_ ≤ 5, are identical to the first five sample singular value vectors *x*_*ℓ*_3_,*j*_, 1 ≤ *ℓ*_3_ ≤ 5. The Pearson correlations between these five loadings and singular value vectors are −0.94, −0.91, 0.88, −0.97 and −0.97 (Here the signs do not mean anything), respectively. It is also shown the regression analysis of the first miRNA PC loading $v_{\ell_{2}=1,j}^{\prime }$ with the top five mRNA PC loadings, *v*_*ℓ*_1_,*j*_, 1 ≤ *ℓ*_1_ ≤ 5,

$$\begin{array}{ccc} v_{\ell_{2}=1,j}^{\prime } & = & C_{0}+\sum_{\ell_{1}=1}^{5}C_{\ell_{1}}v_{\ell_{1},j},\;\;j=1,\ldots ,M \end{array}$$

covers more than 40% of the first miRNA PC loading $v_{\ell_{2}=1,j}^{\prime }$. Since the dimension of vector is *M* = 161, only five components that can cover this amount is highly significant. This suggests that TD can easily decompose single miRNA PC loadings $v_{\ell_{2}=1,j}^{\prime }$ into five mRNA PC loading *v*_*ℓ*_1_,*j*_, 1 ≤ *ℓ*_1_ ≤ 5. The result is that the first five sample singular value vectors (*x*_*ℓ*_3_,*j*_, 1 ≤ *ℓ*_3_ ≤ 5), are almost identical to the first five mRNA PC loadings (*v*_*ℓ*_1_,*j*_, 1 ≤ *ℓ*_3_ ≤ 5). Thus, TD based unsupervised FE can decompose the first dominant miRNA PC loading into five basic (orthogonal) mRNA PC loading. This result is analogous to that seen in the application to synthetic dataset (Fig 4).

It is also important to show that the five mRNA PC loadings *v*_*ℓ*_1_,*j*_, 1 ≤ *ℓ*_1_ ≤ 5 (or the first five sample singular value vectors $x_{\ell_{3},j}^{\text{mRNA}},1\leq \ell_{3}\leq 5$) are distinct also from biological point of view. To demonstrate this, five sets of outliner mRNA probes (associated mRNAs are in S2 Table) were selected using each of the first five mRNA singular value vectors ($x_{\ell_{1},i_{1}}^{\text{mRNA}},1\leq \ell_{1}\leq 5$) each of which is coincident with the first five sample singular value vectors, ($x_{\ell_{3},j}^{\text{mRNA}},1\leq \ell_{3}\leq 5$)as *G*(*ℓ*_1_, *ℓ*_2_, *ℓ*_3_)s with 1 ≤ *ℓ*_1_ = *ℓ*_3_ ≤ 5 have larger absolute values (Table 1). GO (biological process) BP term enrichments were tested by uploading S2 Table to g:Cocoa in g:profiler [27] (S2 File). It is obvious that five sets of mRNAs identified as outliers using each of the first five mRNA singular value vectors are biologically distinct from one another.

### Methodological discussion of TD based unsupervised FE applied to identification of temporally differentially expressed genes

At certain time points in EGF treatment experiments, there is only one measurement, which prevents the application of some statistical methods. Therefore, only vaccination samples were considered. Again, SAM, limma, RF and PCA based unsupervised FE were considered. Here, the samples are assumed to be classified into five time points times three treatments (P, D, NP) equalling 15 classes. Results are shown in Table 5. As with multi-omics data, SAM, limma, and RF failed to identify sufficiently small numbers. This is possibly due to the fact that there are 15 classes. Since even the detection of differences between pairs of any two of the 15 classes can effect results, there are many genes identified as having significant differences. On the other hand, 18 mRNA probes were identified by PCA based unsupervised FE with considering common set when separately applied to three gene expression profiles, $x_{i,t_{1}}^{\text{P}}$ $x_{i,t_{2}}^{\text{D}}$ and $x_{i,t_{3}}^{\text{NP}}$. Thus, relatively successful.

In order to determine biological significance, a reduced number of gene sets was uploaded to MSigDB. Similar to the multi-omics case, SAM indicated too many mRNAs with adjusted *P*-value = 0, therefore no reduced sets could be generated. Two 300 top ranked mRNA probes sets were generated from limma and RF and associated mRNAs were uploaded to MSigDB together with the genes associated with 18 mRNA probes obtained by PCA based unsupervised FE. Within top 10 ranked significant genes set, no vaccination related genes sets were identified for limma or RF. PCA based supervised FE has only two vaccination related genes sets. GSE13485_X_VS_Y_YF17D_VACCINE_PBMC_DN, (X, Y) = (DAY3, DAY7) and (DAY1, DAY7), were identified as the second (adjusted *P* = 1.86 × 10^−6^) and the fourth (adjusted *P* = 4.30 × 10^−5^) significant genes sets, which were smaller than those identified when TD was applied to type II tensors, which identified five gene sets associated with vaccination out of six top ranked gene sets.

Thus, one of four tested methods, PCA based unsupervised FE, could produce some significant results which were inferior to those produced by TD based unsupervised FE applied to type II tensors. As a result, TD based unsupervised FE proved more effective than the other methodologies analysed above.

### Comparison with HO GSVD

To my knowledge, although there are no methods that comprise a tensor from multiple matrices and applies TD to it, similar trials aiming integration of multiple matrices exist. For example, higher order generalized singular value decomposition (HO GSVD) [25] is one such method. Although HO GSVD does not generate tensor, the outcome is quite similar; a set of feature singular value vectors and a unique (common) sample singular value vector which is equivalent to what TD applied to type I tensors generated from Case I data produces. Although Ponnapalli et al [25] employed the distinct terminology from the present study, I continue to use my own terminology in this subsection to avoid confusion.

**Fig 9:**
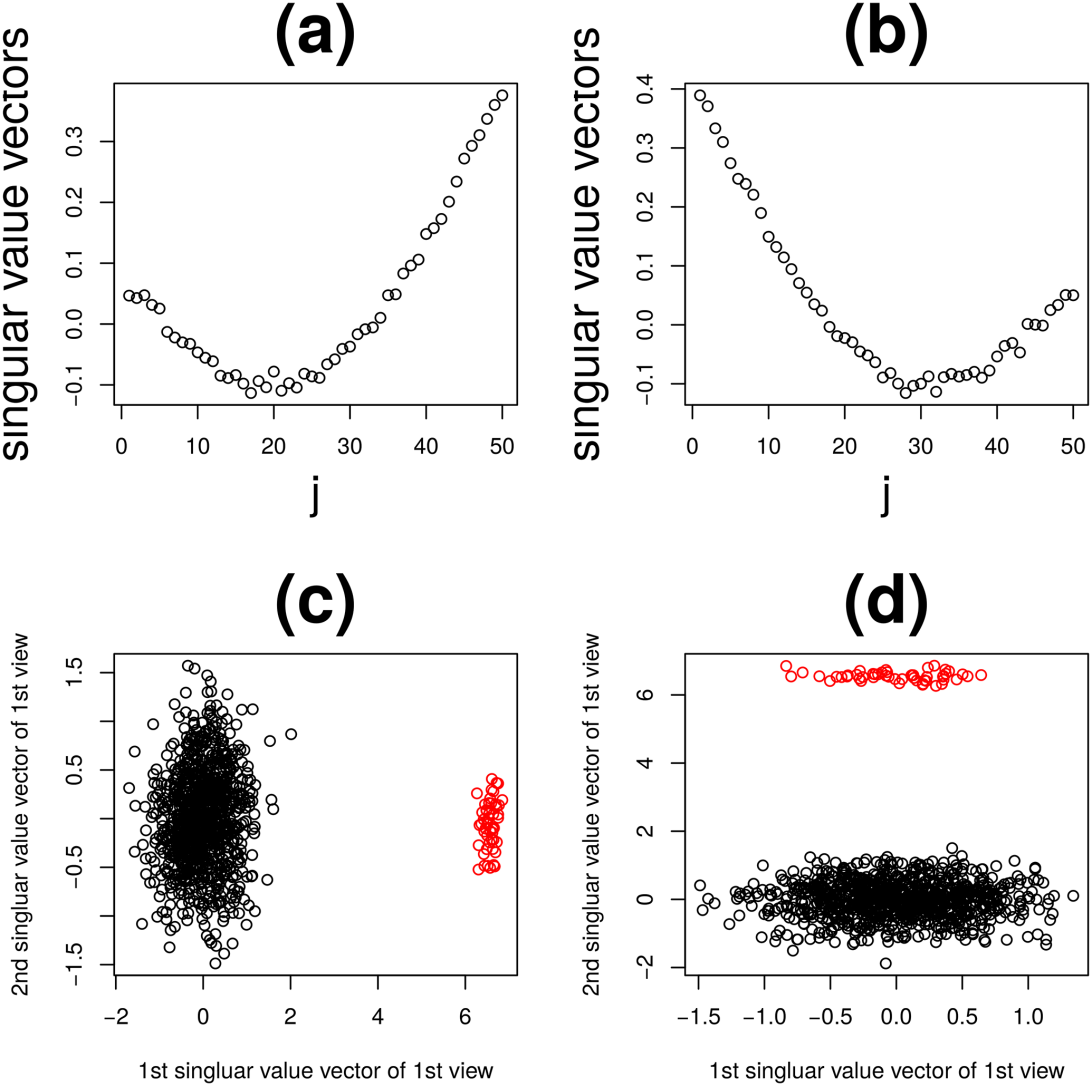
The results of HO GSVD applied to the synthetic data. Red open circles are features with latent correlation (1 ≤ *j* ≤ *N*_0_). The first (a) and the second (b) sample singular value vectors and the first vs the second feature singular value vectors of the first (c) and the second views (d).

First, HO GSVD was applied to synthetic data. Fig 9(a)–9(d) shows the results. Its outcome is close to that when PCA based unsupervised FE was applied to dataset (Fig 8(e)–8(h)). It is in some sense reasonable, since HO GSVD is essentially PCA excluding the fact multiple views share the unique sample singular value matrix, *V*, where the first the second column vectors correspond to the first PC loading of the first and the second views, respectively.

Next, HO GSVD was applied to multi-omics data. Coincidence between sample singular value vectors and class labeling is shown in Fig 1(d). Although four out of five vectors are significantly coincident with class labeling, significance was substantially less than when TD was applied to type I and II tensors, as the *P*-values were larger. Thus, HO GSVD can perform well but is less effective than TD applied to type I or II tensors.

Next, 374 mRNA probes identified as outliers using the first five mRNA feature singular value vectors were selected (associated mRNAs are shown in S1 Table). Uploading the mRNAs associated with 374 mRNA probes to MSigDB, I found that only five out of ten top ranked significant genes sets were related to breast cancer (Table 6), while eight out of ten were related to breast cancer when TD was applied to type I or II tensors (Table 2).

Fifteen miRNAs(miR-127-5p/128/181a/190a/301a/30e*/339-5p/340/361-5p/365 /452/454/455-5p/874/135a) identified as outliers using the first and the second miRNA feature singular value vectors were selected. None were reported in the original study ([30], Table 1). Uploading 15 miRNAs to DIANA-mirpath, I found that “MiRNAs in cancer”, which was the top ranked when TD was applied to type I or type II tensors (Table 3), was not included in the top ten ranked KEGG pathways.

HO GSVD cannot be applied to identification of temporally differentially expressed genes, since HO GSVD can be applied only to Case I data where samples are shared between multiple views.

These slightly poorer outcomes of HO GSVD than TD applied to type I or II tensors suggest the usefulness of tensors when analysing multi-view datasets.

### Biological validations of mRNAs identified in multi-omics data analysis

In the previous subsection, TD based unsupervised FE applied to product of multi-omica profile matrices was validated chiefly from the methodological perspective, and validated partially from the biological perspective.

In this subsection, I try to validate outcomes biologically in more detail, mainly based upon the consideration from oncology.

The samples analysed are essentially proposing the comparison between tumors with and without metastasis. Thus, it is expected that selected genes are mainly related to cancer oncogenesis related to metastasis.

Since Farazi et al [30] who produced the original study, mainly discuss the aberrant expression of miRNAs among samples, there is no in depth discussion about the role of miRNA/mRNA in metastasis. However, as can be seen in the below, much can be discussed from their dataset.

In order to biologically investigate a set of mRNAs identified when type I tensors were considered, to the mRNAs were uploaded to g:profiler (see S3 Table). A large number of enrichments of biological terms were identified.

For example, in GO BP terms, “leukocyte activation” (GO:0045321) was enriched. It was reported to be related to metastasis. Ihnen et al [38] reported a tumor biological context of activated leukocyte cell adhesion molecules (ALCAM) for the development of metastases in breast cancer. Strell et al [39] concluded that the first two steps of the extravasation of tumor cells and leukocytes, rolling and adhesion, seem to have similarities with regards to the mechanisms and receptors involved. King et al [40] identified ALCAM in metastasis of breast cancer cells to the lung. These suggested that metastasis was mediated by the extravasation similar to that of leukocytes. In relaton to this, “positive regulation of leukocyte chemotaxis” (GO:0002690) was also enriched. Wu [41] reported the role of chemotaxis in cell migration. Gradient of chemotaxis mediates cell migration, and possibly metastasis, too.

In GO (cellular component) CC terms, “extracellular region” (GO:0005576) was enriched. Cho et al [42] reported that Herceptin binds to the juxtamembrane region of HER2, identifying this site as a target for anticancer therapies, while overexpression of HER2 is found in 20-30% of human breast cancers, and correlates with more aggressive tumours and a poorer prognosis. It is also primary biomarker of breast cancer in the original study [30]. More generally, Versteeg et al [43] suggested the importance of extracellular signaling pathway in cancer metastasis. It mediates blood vessel wall damage, which may allow tumours to migrate through blood vessels.

In GO (molecular function) MF terms, “CXCR3 chemokine receptor binding” (GO:0048248) was enriched. CXCR3 was reported as a molecular target in breast cancer metastasis [44]; it inhibits tumor cell migration and promotes of host anti-tumour immunity. As suggested in the above, chemotaxis mediates cell migration and chemokine receptor CXCR3 agonist prevents human T-cell migration [45]. Other than in relation to metastasis, inhibition of CXCR3 was also known to mediate tumor growth [46]. “RAGE receptor binding” (GO:0050786) was also enriched. RAGE was reported to mediate tumor progression and metastasis through binding to S100A7 by modulating the tumor microenvironment [47]. It recruits MMP9-positive tumor-associated macrophages and mediates cell migrations.

Other than in GO terms enrichment, transcription factor (TF) SOX9 target genes were enriched. The relation between SOX9 and metastasis was pointed out by many papers. Got et al [48] reported that co-expression of Slug and Sox9 promotes the tumorigenic and metastasis-seeding abilities of human breast cancer cells. SOX9 protein, which is normally nuclear, was instead localized in the cytoplasm of 25-30% invasive ductal carcinomas (IDCs) and lymph node metastases [49]. Lei et al [50] also suggested that Sox9 expression is related to breast cancer metastasis. Although the above are all related to breast cancer, Sox9 was frequently reported to be related to metastasis in various other cancers.

KEGG pathway “Primary immunodeficiency” (KEGG:05340) was also enriched. Development of cancer in patients with primary immunodeficiencies was reported [51]. Monozygotic twin brothers with primary immunodeficiency presented with metastatic adenocarcinoma of unknown primary [52].

In conclusion, our method and strategy correctly identified many cancer related biological terms/concept enrichments, especially metastasis in breast cancer, which is coincident with the purpose of the original study that did not produce results produced here.

### Biological validations of miRNAs identified in multi-omics data analysis

As the relation between mRNAs identified and breast cancer metastasis can be shown, it is necessary to demonstrate the relationship between the miRNAs identified and breast cancer metastasis. Research of the litertature showsthat all seven miRNAs identified when type I tensors were considered(let-7b [53], miR-125b [54–56], miR-143 [57–64], miR-145 [61, 62, 65–68], miR-21 [69–73], miR-22 [74–78] and miR-99a [32, 79]), were reported to be related to metastasis.

Although not all are strictly related to breast cancer, all seven miRNAs identified are frequently reported to be related to metastasis.

## Conclusion

In this paper, a new strategy aiming at multi-view data processing that makes use of tensors generated from multi-view matrices products was proposed. As tensors can be generated from individual measurements, observation under combined conditions, which is generally required to produce tensors from datasets, is not necessary. FEs were performed using singular value vectors generated from TD and biological feasibility was confirmed via comparisons with previously generated annotated gene expression profiles. As this strategy is not restricted to gene expression, its application to other datasets is feasible.

## Supporting information

S1 File Flow chart and variables. A Work flow chart and list of the variables introduced are in S1 File.
(PDF)

S2 File BP term enrichment. BP term enrichments by uploading S2 Table to g:Cocoa in g:profiler.
(PDF)

S1 Table List of genes. Full lists of genes selected by various methods.
(XLSX)

S2 Table List of genes. Lists of genes associated with five gene singular value vectors.
(CSV)

S3 Table Output from g:profiler. Enriched terms when list of genes were uploaded to g profiler.
(XLSX)
