# Correction: Tensor decomposition-based unsupervised feature extraction applied to matrix products for multi-view data processing

**DOI:** 10.1371/journal.pone.0200451

**Published:** 2018-07-18

**Authors:** Y-h. Taguchi

The image for Fig 1 is incorrectly duplicated from Fig 4. Please view the correct Fig 1 here.

**Fig 1:**
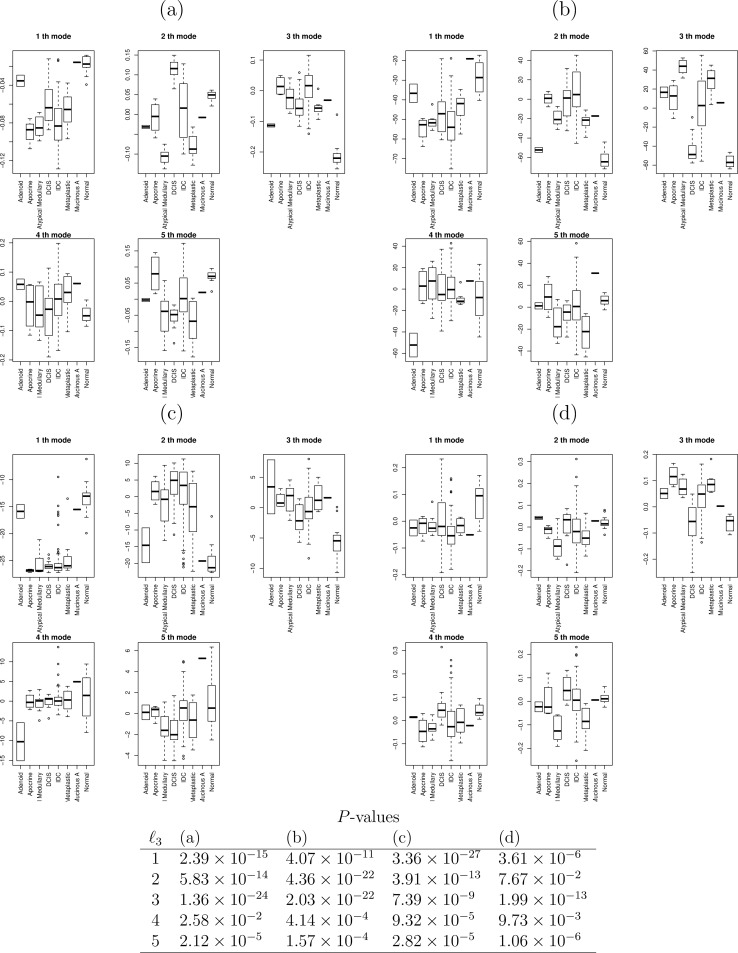
Boxplots of sample singular value vectors x_ℓ3,j_ (a) when TD was applied to the type I tensor and ${\tilde{x}}_{l_{3},j}^{mRNA}$ (b), ${\tilde{x}}_{l_{3},j}^{miRNA}$ (c), 1 ≤ ℓ_3_ ≤ 5, when TD was applied to the type II tensor, generated from mRNA and miRNA expression profiles of multi-omics datasets. (d) Sample singular value vectors when HO GSVD was applied to multi-omics datasets. *P*-values computed by categorical regression attributed to (a) to (d) were below the figures.

There are errors in the second paragraph of the subsection titled, “Definition and terminology of TD” in the Materials and Methods section. All instances of the following, “*G*(*n*_1_, *n*_2_, …, *n*_m_)” should instead read as, $“G(l_{1},l_{2},\ldots ,l_{m})$. The correct paragraph should be: TD is the expansion of tensor $x_{n_{1},n_{2},\ldots ,n_{m}}$, *n*_*k*_ = 1, …, *N*_*k*_, 1 ≤ *k* ≤ *m* in the form

$$x_{n_{1},n_{2},\ldots ,n_{m}}=\sum_{l_{1}=1}^{N_{1}}\ldots \sum_{l_{m}=1}^{N_{m}}G(l_{1},l_{2},\ldots ,l_{m})\prod_{k=1}^{m}x_{n_{k},l_{k}}$$

where $x_{n_{k},l_{k}}$, 1 ≤ *k* ≤ *m*, are orthogonal matrices. Since $x_{n_{1},n_{2},\ldots ,n_{m}}$ is as large as $G(l_{1},l_{2},\ldots ,l_{m})$, this formula is clearly overcomplete and does not give unique expansion. In this study, in order to decide $G(l_{1},l_{2},\ldots ,l_{m})$, $x_{n_{k},l_{k}}$, 1 ≤ *k* ≤ *m* uniquely, I employ the higher order singular value decomposition (HOSVD) algorithm [23], which has successfully used to analyse microarrays [24] previously. $G(l_{1},l_{2},\ldots ,l_{m})$ is a core matrix. $x_{n_{k},l_{k}}$, 1 ≤ *k* ≤ *m*, are singular value matrices and their column vectors are singular value vectors. $G(l_{1},l_{2},\ldots ,l_{m})$, having larger absolute values, has more contribution to $x_{n_{1},n_{2},\ldots ,n_{m}}$. Since the combination of $x_{n_{k},l_{k}}$, 1 ≤ *k* ≤ *m*, associated with $G(l_{1},l_{2},\ldots ,l_{m})$ to which larger absolute values were attributed contributes more collectively to $x_{n_{1},n_{2},\ldots ,n_{m}}$, they are more likely to be associated with one another.
