# Supplementary material for: Tensor decomposition-based unsupervised feature extraction applied to matrix products for multi-view data processing
Source: PLoS One. 2017 Aug 25;12(8):e0183933. doi: 10.1371/journal.pone.0183933 (PMC5571984; doi:10.1371/journal.pone.0183933)
Supplement: S1 File — A Work flow chart and list of the variables introduced are in S1 File. (PDF) [file pone.0183933.s001.pdf]

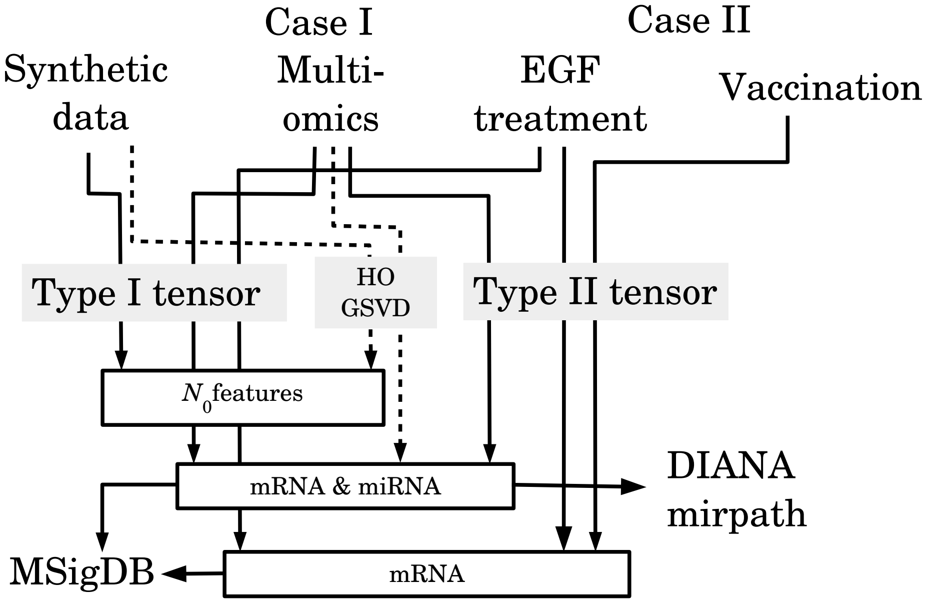

	raw data	type I tensor	type II tensor
Synthetic data (case I)	$x^{(k)}_{i_k,j}$	$x_{i_1,i_2,j}$<br>$x^{(1)}_{\ell_1,i_1}, x^{(2)}_{\ell_2,i_2}, x_{\ell_3,j}$	$\tilde{x}_{i_1,i_2}$<br>$\tilde{x}^{(1)}_{\ell_1,i_1}, \tilde{x}^{(2)}_{\ell_2,i_2}, \tilde{x}^{(1)}_{\ell_3,j}, \tilde{x}^{(2)}_{\ell_3,j}$
		$1 \leq i_1, i_2, \ell_1, \ell_2 \leq N(=1000), 1 \leq j, \ell_3 \leq M(=50)$	
Multi-omics (case I)	$x^{\text{mRNA}}_{i_1,j}, x^{\text{miRNA}}_{i_2,j}$	$x_{i_1,i_2,j}$<br>$x^{\text{mRNA}}_{\ell_1,i_1}, x^{\text{miRNA}}_{\ell_2,i_2}, x_{\ell_3,j}$	$\tilde{x}_{i_1,i_2}$<br>$\tilde{x}^{\text{mRNA}}_{\ell_1,i_1}, \tilde{x}^{\text{miRNA}}_{\ell_2,i_2}, \tilde{x}^{\text{mRNA}}_{\ell_3,j}, \tilde{x}^{\text{miRNA}}_{\ell_3,j}$
		$1 \leq i_1, \ell_1 \leq 13393,\ 1 \leq i_2, \ell_2 \leq 755,\ 1 \leq j, \ell_3 \leq 161$	
EGF treatment (case II)	$x^{\text{control}}_{i,t_1}, x^{\text{EGF}}_{i,t_2}$	$x_{t_1,t_2,j}$<br>$x^{\text{control}}_{\ell_1,t_1}, x^{\text{EGF}}_{\ell_2,t_2}, x_{\ell_3,i}$	$\tilde{x}_{t_1,t_2}$<br>$\tilde{x}^{\text{control}}_{\ell_1,t_1}, \tilde{x}^{\text{EGF}}_{\ell_2,t_2}, \tilde{x}^{\text{control}}_{\ell_3,i}, \tilde{x}^{\text{EGF}}_{\ell_3,i}$
		$1 \leq t_1, \ell_1 \leq T_{\text{control}}(=14),\ 1 \leq t_2, \ell_2 \leq T_{\text{EGF}}(=14),\ 1 \leq i, \ell_3 \leq 39937$	
Vaccination (case II)	$x^{\text{P}}_{i,t_1}, x^{\text{D}}_{i,t_2}, x^{\text{NP}}_{i,t_3}$	—	$\tilde{x}_{t_1,t_2,t_3}$<br>$\tilde{x}^{\text{P}}_{\ell_1,t_1}, \tilde{x}^{\text{D}}_{\ell_2,t_2}, \tilde{x}^{\text{NP}}_{\ell_3,t_3}, \tilde{x}^{\text{P}}_{\ell_4,i}, \tilde{x}^{\text{D}}_{\ell_4,i}, \tilde{x}^{\text{NP}}_{\ell_4,i}$
	$1 \leq t_1, \ell_1 \leq T_{\text{P}}(=58),\ 1 \leq t_2, \ell_2 \leq T_{\text{D}}(=52),\ 1 \leq t_3, \ell_3 \leq T_{\text{NP}}(=72),\ 1 \leq i, \ell_4 \leq 22277$		
