# Supplementary material for: Tensor decomposition-based unsupervised feature extraction applied to matrix products for multi-view data processing
Source: PLoS One. 2017 Aug 25;12(8):e0183933. doi: 10.1371/journal.pone.0183933 (PMC5571984; doi:10.1371/journal.pone.0183933)
Supplement: S2 File — BP term enrichments by uploading S2 Table to g:Cocoa in g:profiler. (PDF) [file pone.0183933.s002.pdf]

source	term name<br>Gene Ontology (Biological process)	term ID	n. of term genes	corrected p-value	1	2	3	4	5
BP	tumor necrosis factor superfamily cytokine production	GO:0071706	106	4.00e-02					5
BP	tumor necrosis factor production	GO:0032640	101	3.16e-02					5
BP	regulation of tumor necrosis factor superfamily cytokine production	GO:1903555	103	3.48e-02					5
BP	regulation of tumor necrosis factor production	GO:0032680	100	3.01e-02					5
BP	response to gamma radiation	GO:0010332	50	2.34e-02			4		
BP	positive regulation of cytosolic calcium ion concentration	GO:0007204	265	3.52e-02				8	
BP	programmed cell death	GO:0012501	1812	2.06e-02				21	
BP	apoptotic process	GO:0006915	1791	1.72e-02				21	
BP	negative regulation of multicellular organism growth	GO:0040015	12	4.06e-02				3	
BP	mammary gland development	GO:0030879	136	5.67e-04	8				
BP	phagocytosis, recognition	GO:0006910	22	5.11e-03				4	
BP	animal organ morphogenesis	GO:0009887	990	3.02e-02	16				
BP	sequestering of metal ion	GO:0051238	127	3.79e-02				6	
BP	mammary gland epithelial cell proliferation	GO:0033598	28	1.95e-02	4				
BP	phagocytosis, engulfment	GO:0006911	38	4.91e-02				4	
BP	single-organism metabolic process	GO:0044710	4155	4.38e-03					29
BP	homeostatic process	GO:0042592	1629	1.54e-02				20	
BP	ion homeostasis	GO:0050801	716	1.64e-02				13	
BP	inorganic ion homeostasis	GO:0098771	654	6.11e-03				13	
BP	cation homeostasis	GO:0055080	639	4.73e-03				13	
BP	divalent inorganic cation homeostasis	GO:0072507	431	4.61e-04				12	
BP	metal ion homeostasis	GO:0055065	557	1.01e-03				13	
BP	calcium ion homeostasis	GO:0055074	399	1.39e-02				10	
BP	cellular chemical homeostasis	GO:0055082	721	1.77e-02				13	
BP	cellular ion homeostasis	GO:0006873	583	1.70e-03				13	
BP	cellular cation homeostasis	GO:0030003	568	1.26e-03				13	
BP	cellular divalent inorganic cation homeostasis	GO:0072503	412	2.83e-04				12	
BP	cellular metal ion homeostasis	GO:0006875	489	2.27e-04				13	
BP	cellular calcium ion homeostasis	GO:0006874	387	1.06e-02				10	
BP	tissue development	GO:0009888	1769	5.24e-03			17		
BP	epithelium development	GO:0060429	1072	9.39e-03			13		
BP	tissue morphogenesis	GO:0048729	629	1.43e-02			10		
BP	morphogenesis of an epithelium	GO:0002009	528	2.55e-02			9		
BP	immune system process	GO:0002376	2387	1.35e-24				51	21
BP	immune effector process	GO:0002252	713	1.35e-10			10	22	
BP	leukocyte mediated immunity	GO:0002443	332	2.80e-04				11	
BP	lymphocyte mediated immunity	GO:0002449	262	3.04e-04				10	
BP	signaling	GO:0023052	6132	6.03e-07				52	
BP	single organism signaling	GO:0044700	6120	5.56e-07				52	
BP	biological regulation	GO:0065007	11372	6.40e-03				65	
BP	regulation of biological process	GO:0050789	10756	6.19e-03				63	
BP	positive regulation of biological process	GO:0048518	5194	3.52e-04				43	
BP	positive regulation of signaling	GO:0023056	1539	2.60e-02				19	
BP	regulation of immune system process	GO:0002682	1336	5.08e-13			14	31	
BP	positive regulation of immune system process	GO:0002684	934	4.22e-11				25	
BP	regulation of cellular process	GO:0050794	10218	5.46e-04				63	
BP	regulation of cell adhesion	GO:0030155	621	2.10e-02				12	
BP	response to stimulus	GO:0050896	8162	1.72e-13				68	
BP	response to chemical	GO:0042221	4059	1.43e-04				38	
BP	response to organic substance	GO:0010033	2799	1.49e-06				34	23
BP	response to cytokine	GO:0034097	793	8.20e-14				26	
BP	response to interleukin-1	GO:0070555	114	2.04e-02				6	
BP	response to tumor necrosis factor	GO:0034612	276	4.25e-05				11	
BP	response to lipid	GO:0033993	870	2.20e-02					12
BP	response to biotic stimulus	GO:0009607	836	2.95e-13				26	
BP	response to external stimulus	GO:0009605	2021	6.24e-09			17	32	18
BP	response to external biotic stimulus	GO:0043207	794	1.01e-12				25	
BP	response to other organism	GO:0051707	793	9.78e-13				25	
BP	response to virus	GO:0009615	305	7.96e-07				13	
BP	response to bacterium	GO:0009617	516	5.90e-06				15	
BP	regulation of response to stimulus	GO:0048583	3592	1.77e-05				37	
BP	regulation of response to external stimulus	GO:0032101	703	2.29e-03				14	11
BP	response to stress	GO:0006950	3579	7.15e-09			23	42	28
BP	defense response	GO:0006952	1448	4.16e-19			14	38	22
BP	regulation of defense response	GO:0031347	658	6.53e-03				13	
BP	inflammatory response	GO:0006954	656	1.62e-06				12	15
BP	defense response to other organism	GO:0098542	465	1.89e-14				22	
BP	defense response to bacterium	GO:0042742	239	9.52e-06				11	
BP	defense response to virus	GO:0051607	227	7.90e-05				10	
BP	response to wounding	GO:0009611	644	4.95e-02					10
BP	immune response	GO:0006955	1530	9.36e-29			15	47	
BP	humoral immune response	GO:0006959	223	9.92e-03				8	
BP	adaptive immune response	GO:0002250	390	2.82e-12				19	
BP	adaptive immune response based on somatic recombination of immune ...	GO:0002460	262	2.48e-05				11	
BP	innate immune response	GO:0045087	791	7.71e-14				26	12
BP	response to interferon-gamma	GO:0034341	155	2.00e-06				10	
BP	response to type I interferon	GO:0034340	82	2.96e-03				6	
BP	regulation of immune response	GO:0050776	879	1.06e-10				24	
BP	cellular response to stimulus	GO:0051716	6818	5.64e-07				55	
BP	cellular response to chemical stimulus	GO:0070887	2643	7.12e-06	29			32	
BP	cellular response to organic substance	GO:0071310	2187	3.45e-05				28	
BP	cellular response to cytokine stimulus	GO:0071345	697	6.16e-13				24	
BP	cellular response to tumor necrosis factor	GO:0071356	258	2.64e-04				10	
BP	cellular response to type I interferon	GO:0071357	78	2.20e-03				6	
BP	cellular response to interferon-gamma	GO:0071346	133	9.88e-06				9	
BP	positive regulation of response to stimulus	GO:0048584	1994	4.33e-09				32	
BP	positive regulation of response to external stimulus	GO:0032103	264	2.68e-05				11	
BP	positive regulation of chemotaxis	GO:0050921	118	2.48e-02				6	
BP	positive regulation of defense response	GO:0031349	399	1.76e-03				11	
BP	positive regulation of immune response	GO:0050778	664	3.71e-08				19	
BP	activation of immune response	GO:0002253	536	9.88e-06				15	
BP	multicellular organismal process	GO:0032501	6896	1.84e-02		37			
BP	single-multicellular organism process	GO:0044707	5900	1.00e-03		36			36
BP	regulation of multicellular organismal process	GO:0051239	2631	4.77e-04				29	21
BP	regulation of localization	GO:0032879	2442	4.16e-02					20
BP	cell communication	GO:0007154	6147	6.66e-07				52	
BP	signal transduction	GO:0007165	5674	1.17e-07				51	
BP	cell surface receptor signaling pathway	GO:0007166	2661	3.93e-10				38	
BP	cytokine-mediated signaling pathway	GO:0019221	542	6.64e-12				21	
BP	chemokine-mediated signaling pathway	GO:0070098	80	2.56e-03				6	
BP	type I interferon signaling pathway	GO:0060337	78	2.20e-03				6	
BP	interferon-gamma-mediated signaling pathway	GO:0060333	80	2.56e-03				6	
BP	immune response-regulating signaling pathway	GO:0002764	513	4.84e-05				14	
BP	immune response-regulating cell surface receptor signaling pathway	GO:0002768	380	1.08e-03				11	
BP	immune response-activating signal transduction	GO:0002757	482	2.20e-05				14	
BP	immune response-activating cell surface receptor signaling pathway	GO:0002429	350	4.77e-04				11	
BP	antigen receptor-mediated signaling pathway	GO:0050851	209	2.32e-06			6	11	
BP	B cell receptor signaling pathway	GO:0050853	53	5.37e-06				7	
BP	single organism cell adhesion	GO:0098602	760	2.22e-05				17	
BP	positive regulation of cellular process	GO:0048522	4635	4.86e-04				40	
BP	positive regulation of cell communication	GO:0010647	1532	2.44e-02				19	
BP	leukocyte proliferation	GO:0070661	268	3.76e-04				10	
BP	regulation of leukocyte proliferation	GO:0070663	198	4.64e-02				7	
BP	mononuclear cell proliferation	GO:0032943	254	2.28e-04				10	
BP	regulation of mononuclear cell proliferation	GO:0032944	190	3.55e-02				7	
BP	cell-cell adhesion	GO:0098609	1145	7.58e-03				17	
BP	single organismal cell-cell adhesion	GO:0016337	708	5.72e-05				16	
BP	leukocyte cell-cell adhesion	GO:0007159	469	1.60e-06				15	
BP	leukocyte aggregation	GO:0070486	436	5.82e-07				15	
BP	lymphocyte aggregation	GO:0071593	429	4.89e-05				13	
BP	neutrophil aggregation	GO:0070488	2	2.07e-02				2	2
BP	regulation of cell-cell adhesion	GO:0022407	371	7.30e-03				10	
BP	regulation of leukocyte cell-cell adhesion	GO:1903037	298	9.62e-03				9	
BP	cell activation	GO:0001775	872	8.77e-12				25	
BP	leukocyte activation	GO:0045321	709	1.08e-11				23	
BP	myeloid leukocyte activation	GO:0002274	155	9.31e-03				7	
BP	myeloid dendritic cell activation	GO:0001773	29	1.63e-02				4	
BP	lymphocyte activation	GO:0046649	606	4.84e-12				22	
BP	B cell activation	GO:0042113	228	5.81e-06				11	
BP	lymphocyte proliferation	GO:0046651	252	2.11e-04				10	
BP	T cell aggregation	GO:0070489	428	4.76e-05				13	
BP	T cell activation	GO:0042110	428	4.76e-05				13	
BP	regulation of cell activation	GO:0050865	470	1.56e-07				16	
BP	regulation of leukocyte activation	GO:0002694	436	5.09e-08				16	
BP	regulation of lymphocyte activation	GO:0051249	380	6.43e-09				16	
BP	regulation of lymphocyte proliferation	GO:0050670	189	3.43e-02				7	
BP	regulation of B cell activation	GO:0050864	112	5.06e-05			6	8	
BP	regulation of T cell activation	GO:0050863	283	6.30e-03				9	
BP	positive regulation of cell activation	GO:0050867	291	4.46e-07				13	
BP	positive regulation of leukocyte activation	GO:0002696	282	3.02e-07				13	
BP	positive regulation of lymphocyte activation	GO:0051251	258	1.53e-06				12	
BP	positive regulation of B cell activation	GO:0050871	75	1.74e-03			5	6	
BP	leukocyte migration	GO:0050900	370	8.75e-05				12	9
BP	mononuclear cell migration	GO:0071674	73	1.48e-03				6	
BP	regulation of mononuclear cell migration	GO:0071675	34	3.13e-02				4	
BP	myeloid leukocyte migration	GO:0097529	159	7.74e-04				8	
BP	granulocyte migration	GO:0097530	111	1.75e-02				6	5
BP	lymphocyte migration	GO:0072676	80	2.56e-03				6	
BP	T cell migration	GO:0072678	38	4.91e-02				4	
BP	regulation of lymphocyte migration	GO:2000401	38	4.91e-02				4	
BP	regulation of T cell migration	GO:2000404	25	8.76e-03				4	
BP	leukocyte migration involved in inflammatory response	GO:0002523	13	1.70e-02					3
BP	positive regulation of leukocyte migration	GO:0002687	106	1.34e-02				6	
BP	positive regulation of lymphocyte migration	GO:2000403	25	8.76e-03				4	
BP	positive regulation of T cell migration	GO:2000406	20	3.41e-03				4	
BP	positive regulation of mononuclear cell migration	GO:0071677	19	2.74e-03				4	
BP	positive regulation of inflammatory response	GO:0050729	115	2.14e-02				6	
BP	cell chemotaxis	GO:0060326	246	1.69e-04				10	
BP	leukocyte chemotaxis	GO:0030595	187	1.93e-04				9	
BP	lymphocyte chemotaxis	GO:0048247	53	2.13e-04				6	
BP	T cell chemotaxis	GO:0010818	18	2.17e-03				4	
BP	granulocyte chemotaxis	GO:0071621	99	8.98e-03				6	5
BP	monocyte chemotaxis	GO:0002548	58	3.70e-04				6	
BP	regulation of leukocyte chemotaxis	GO:0002688	96	7.50e-03				6	
BP	regulation of lymphocyte chemotaxis	GO:1901623	19	2.74e-03				4	
BP	regulation of T cell chemotaxis	GO:0010819	11	2.39e-04				4	
BP	regulation of monocyte chemotaxis	GO:0090025	19	2.74e-03				4	
BP	positive regulation of leukocyte chemotaxis	GO:0002690	79	2.37e-03				6	
BP	positive regulation of monocyte chemotaxis	GO:0090026	14	7.17e-04				4	
BP	positive regulation of T cell chemotaxis	GO:0010820	10	2.23e-02				3	
BP	leukocyte differentiation	GO:0002521	464	7.64e-03				11	
BP	lymphocyte differentiation	GO:0030098	307	1.32e-03				10	
BP	T cell differentiation	GO:0030217	205	5.31e-03				8	
BP	positive regulation of signal transduction	GO:0009967	1408	2.95e-02				18	
BP	interleukin-7-mediated signaling pathway	GO:0038111	2	4.79e-02				2	
BP	positive regulation of intracellular signal transduction	GO:1902533	898	3.91e-02				14	
